# Identification of an Elite Core Panel as a Key Breeding Resource to Accelerate the Rate of Genetic Improvement for Irrigated Rice

**DOI:** 10.1186/s12284-021-00533-5

**Published:** 2021-11-13

**Authors:** Roselyne U. Juma, Jérôme Bartholomé, Parthiban Thathapalli Prakash, Waseem Hussain, John D. Platten, Vitaliano Lopena, Holden Verdeprado, Rosemary Murori, Alexis Ndayiragije, Sanjay Kumar Katiyar, Md Rafiqul Islam, Partha S. Biswas, Jessica E. Rutkoski, Juan D. Arbelaez, Felister N. Mbute, Douglas W. Miano, Joshua N. Cobb

**Affiliations:** 1 Rice Breeding Innovations Platform, International Rice Research Institute, 1301 Los Baños, Metro, DAPO Box 7777, Manila, Philippines; 2 Present Address: Kenya Agricultural and Livestock Research Organization, 50100-169 Kakamega, Kenya; 3 AGAP Institut, CIRAD, INRA, Montpellier SupAgro, Univ Montpellier, Montpellier, France; 4 International Rice Research Institute (IRRI) C/O ILRI, Old Naivasha Road, PO Box 30709, 00100 Nairobi, Kenya; 5 Institiuto de Investigação de Moçambique (IIAM), Av. das FPLM nr 2698, Recinto do IIAM, Maputo, Mozambique; 6 International Rice Research Institute, South Asia Hub, ICRISAT, Hyderabad, 502324 India; 7 Bangladesh Office, International Rice Research Institute (IRRI), Dhaka, Bangladesh; 8 Present Address: Plant Breeding Division, Bangladesh Rice Research Institute (BRRI), Gazipur, Bangladesh; 9 Present Address: University of Illinois at Urbana-Champaign, Urbana, USA Illinois; 10 Department of Plant Science and Crop Protection, University of Nairobi, PO Box 29053, 00625 Kangemi, Kenya; 11 Present Address: RiceTec. Inc, PO Box 1305, Alvin, TX 77512 USA

**Keywords:** Rice, *Oryza sativa*, Genetic gain, Grain yield, Breeding values, Elite panel

## Abstract

Rice genetic improvement is a key component of achieving and maintaining food security in Asia and Africa in the face of growing populations and climate change. In this effort, the International Rice Research Institute (IRRI) continues to play a critical role in creating and disseminating rice varieties with higher productivity. Due to increasing demand for rice, especially in Africa, there is a strong need to accelerate the rate of genetic improvement for grain yield. In an effort to identify and characterize the elite breeding pool of IRRI’s irrigated rice breeding program, we analyzed 102 historical yield trials conducted in the Philippines during the period 2012–2016 and representing 15,286 breeding lines (including released varieties). A mixed model approach based on the pedigree relationship matrix was used to estimate breeding values for grain yield, which ranged from 2.12 to 6.27 t·ha^−1^. The rate of genetic gain for grain yield was estimated at 8.75 kg·ha^−1^ year^−1^ (0.23%) for crosses made in the period from 1964 to 2014. Reducing the data to only IRRI released varieties, the rate doubled to 17.36 kg·ha^−1^ year^−1^ (0.46%). Regressed against breeding cycle the rate of gain for grain yield was 185 kg·ha^−1^ cycle^−1^ (4.95%). We selected 72 top performing lines based on breeding values for grain yield to create an elite core panel (ECP) representing the genetic diversity in the breeding program with the highest heritable yield values from which new products can be derived. The ECP closely aligns with the indica 1B sub-group of *Oryza sativa* that includes most modern varieties for irrigated systems. Agronomic performance of the ECP under multiple environments in Asia and Africa confirmed its high yield potential. We found that the rate of genetic gain for grain yield found in this study was limited primarily by long cycle times and the direct introduction of non-improved material into the elite pool. Consequently, the current breeding scheme for irrigated rice at IRRI is based on rapid recurrent selection among highly elite lines. In this context, the ECP constitutes an important resource for IRRI and NAREs breeders to carefully characterize and manage that elite diversity.

**Supplementary Information:**

The online version contains supplementary material available at 10.1186/s12284-021-00533-5.

## Introduction

Rice *(Oryza sativa L.)* is one of the world’s major staple crops feeding more than 3.5 billion people (Global Rice Science Partnership 2013). It is believed that by 2050 the global population will be approximately 10 billion (United Nations 2019) and much of this population increase will occur in the regions of Africa and Southern Asia, which are highly dependent on rice. As such, rice will be crucial to ensuring equitable food security for the foreseeable future (Peng et al. 2004; Godfray 2014; Li et al. 2018). Challenges posed by climate change as well as increasing consumer demand further highlight the importance of rice to global food security (Silvern and Young 2013). While agricultural intensification using modernized management practices (Garnett et al. 2013) can help boost productivity, the importance of rice genetic improvement in the context of these management systems is also an important driver of sustainable productivity (Guimaraes 2009; Atlin et al. 2017). The rate at which this genetic improvement occurs is often referred to as genetic gain and in order to deliver improved varieties to the farmers of the twenty-first century, the rate of genetic gain in rice must accelerate relative to twentieth century levels (Atlin et al. 2017).

With the acceleration of genotyping technologies through the early twenty-first century and the subsequent maturation of genomic selection-based breeding strategies, there has been a renewed interest in the application of quantitative genetics to plant breeding programs (Cobb et al. 2019b; Bernardo 2020). To this end the irrigated rice breeding program at the International Rice Research Institute (IRRI) has spent significant effort to develop a modernized approach to rice breeding to substantially and sustainably increase response to selection (Collard et al. 2019). In addition to implementing accelerated single seed descent strategies (Collard et al. 2017), another major pillar of IRRI’s effort to transform rice breeding is the deep characterization of the elite genetic base from which new products are derived. While the characterization and dissection of rice genetic diversity in public germplasm collections has advanced considerably (Li et al. 2014; McCouch et al. 2016; Sun et al. 2017), to be fully leveraged for varietal improvement, it needs to be paired with an equally in-depth characterization of the elite genetic diversity residing in breeding programs across the world.

**Fig. 1:**
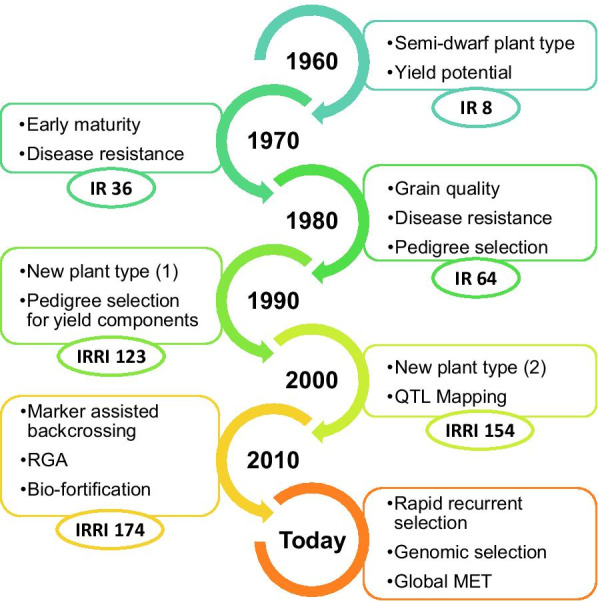
Evolution of main techniques and breeding targets across decades for the irrigated breeding program at IRRI. Changes in breeding objectives in the decade are indicated in the boxes. Important IRRI rice varieties for each decade are shown in the ellipses

The irrigated rice breeding program at IRRI has been a source of elite breeding germplasm for decades (Peng and Khush 2003; Mackill and Khush 2018; Collard et al. 2019). This genetic diversity has been utilized in combination with landraces and local varieties to contribute substantially to the yield improvement achieved in Asia to date. The breeding strategies used to achieve this post-Green Revolution yield improvement however, frequently varied according to funding priorities, available technology, and evolution of scientific thinking (see Fig. 1). IRRI’s early breeding effort culminated in the development of IR8, the first widely-adopted semi-dwarf variety of the Green Revolution (Chandler 1982; Peng et al. 1999; Peng and Khush 2003). Though this variety was high yielding, it lacked acceptable cooking and eating quality and therefore was quickly superseded by other varieties that excelled in both grain yield and marketability (Khush 2001). During this time, a focus on improved disease resistance and continued efforts to increase genetic variation led to many new varieties introgressed with genetics from wild species (Brar and Khush 2002, 2018) that were created using strategies such as backcrossing, top crossing, and pedigree breeding methods. IR 36, for instance, resulted from the combination of 13 landraces from six different countries (Khush 2005). This variety displayed good grain quality, early maturity, tolerance to abiotic stresses, and resistance to multiple pests and disease (Peng and Khush 2003). Further advances in grain quality (soft gel consistency, translucent and long slender grains, intermediate amylose content and intermediate gelatinization temperature) were made with the release of IR 64 which resulted from combining extant improved lines with 19 traditional varieties (Mackill and Khush 2018), but which was still heavily based on IR8. A renewed focus on yield improvement in the late 1980s and 1990s sparked the development of an ideotype breeding strategy known as the new plant type (NPT, Fig. 1) (Cassman 1994; Peng et al. 2004; Yadi et al. 2021). With the advent of the molecular marker technologies during the same period, this was quickly followed up by selection strategies based on marker-assisted backcrossing to introduce major genes for biotic or abiotic stress tolerance to produce enhanced versions of existing varieties. This effort was recently coupled with an enhanced focus on bio-fortification in order to couple high yield with high nutritional value. However, post-Green Revolution breeding for quality and disease resistance, while successful, has not brought about the realized genetic gain for yield that is needed to meet the projected demand. More recent approaches aim to integrate principles of quantitative genetics into the breeding strategy by focusing the molecular breeding strategy on well-known high-value haplotypes and using a genomics-enabled rapid recurrent selection strategy to improve quantitative traits mainly through accelerated breeding cycles (Fig. 1).

The objective of this study was twofold: (i) estimate gains in breeding value for yield over the entire history of IRRI’s breeding program for irrigated systems, and (ii) identify and characterize a panel of elite lines from among the available germplasm that balances high breeding value for yield with sufficient genetic variance to preserve long term gain from selection. To this end, we gathered historical data from 102 yield trials in the IRRI phenotypic database (Breeding4Results 2021) spanning the years from 2012 to 2016 and combined it with pedigree data from the International Rice Information System (McLaren et al. 2005; Collard et al. 2019) to estimate breeding values for grain yield for all extant or recently extant lines. These trials included most of the existing advanced material of the breeding program as well as many replicated observations of IRRI released varieties, allowing us to estimate the rate of genetic gain over five decades. The same data was then used to identify high yielding lines from the breeding program to form the IRRI irrigated elite core panel (ECP). Seventy-two lines were ultimately chosen to comprise the elite panel and were subjected to extensive genetic and phenotypic characterization to assess suitability for short-cycle recurrent selection.

## Results

### Estimation of the Genetic Gain for Grain Yield

**Fig. 2:**
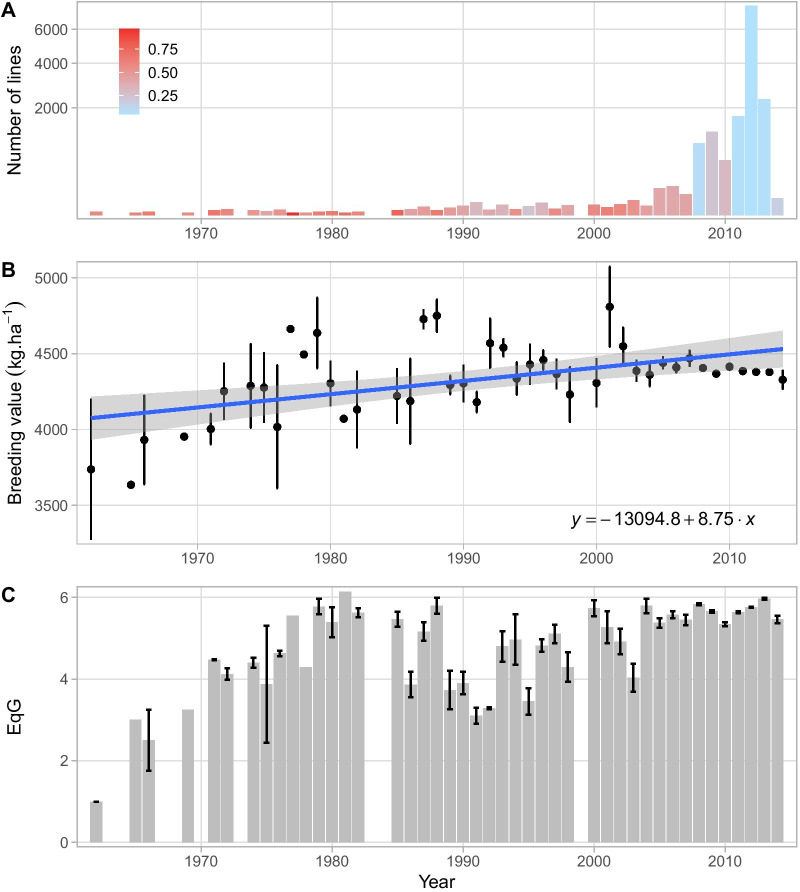
Time trend of important dataset characteristics used to assess genetic gain. **A** Evolution of the number of lines evaluated for this analysis according to the year the cross was initially made. The color gradient indicates the average reliability associated with the breeding values for yield. **B** Time trend in average breeding value for grain yield across years. The error bars represent the standard error associated with the mean. The blue line represents the linear regression of the breeding value for grain yield on the year. **C** Evolution of the average equivalent complete generation (EqG) of the lines through time. The error bars represent the standard error associated with the mean

Genetic gain for grain yield was estimated as a function of change in breeding value over time. Breeding values for 15,286 lines evaluated in 102 trials conducted between 2012 and 2016 were estimated using a two-stage mixed model analysis (Table 1, Additional file 1: Table S1). The majority of these lines were advanced lines from the breeding program that never achieved varietal status and released varieties from different decades. Eighty percent of the lines originated from crosses that were made after 2009 (Fig. 2A). As expected, the reliability of the breeding values of older lines (generated before 2000) were higher compared to more recent material, with an average value of 0.43 (σ = 0.23) and 0.1 (σ = 0.17), respectively. Breeding values for grain yield ranged from 2.12 to 6.27 t·ha^−1^. The genetic trend as measured by this analysis of the IRRI irrigated rice breeding program since its initiation in 1960 to 2014 is presented in Fig. 2B. Over this period the linearized genetic trend was estimated to be 8.75 kg·ha^−1^ year^−1^ (0.23%). Despite the smaller sample size for the earlier historical periods, an upward trend from 1960 to 1980 is apparent followed by a period of variability in the average breeding value which eventually plateaus around 4.38 t·ha^−1^ after 2008. In order to interrogate the drivers of this genetic trend further, the equivalent complete generation (EqG, see “Materials and Methods” section) for each line was calculated as an estimate of the number of effective breeding cycles that had taken place prior to the crossing event. EqG is a key indicator of the rate of introduction of new material and the extent at which improved material is recycled into the breeding program. A similar trend to breeding values was also observed for EqG for the same period. Values had exceeded two by the end of the sixties to reach its maximum average value of six in the eighties (Fig. 2C). This was followed by a marked decrease to an average value of four in the nineties the average maximal values after 2000 never exceeded six equivalent generations. In addition, a large variance in EqG was found across lines from the most recent decade with values ranging from 1 to 7.56 highlighting the extensive use of non-improved material in combination with more elite lines.

**Table 1:** Summary of yield trials used to estimate breeding values for grain yield

Details	Year				
	2012	2013	2014	2015	2016
Number of trials	1	8	11	49	33
Average harvest area (sqm)	4.84	4.49	4.34	4.34	4.58
Number of plots	192	5272	13,822	14,926	13,246
Number of tested lines	64	2487	12,505	10,065	9793
Experimental designs	R	AL, R, RC	AR, R, AU, PR, RC	R, AR, AL, PR, RC, AD	AR, R, PR, RC

These trials were conducted during 2012–2016 for IRRI's rice breeding program for irrigated systems

Experimental design used in the retrieved studies included—R = RCBD, AR = Augmented RCBD, AL = Alpha lattice, PR = P-Rep, RC = Row Column, AD = Augmented

**Fig. 3:**
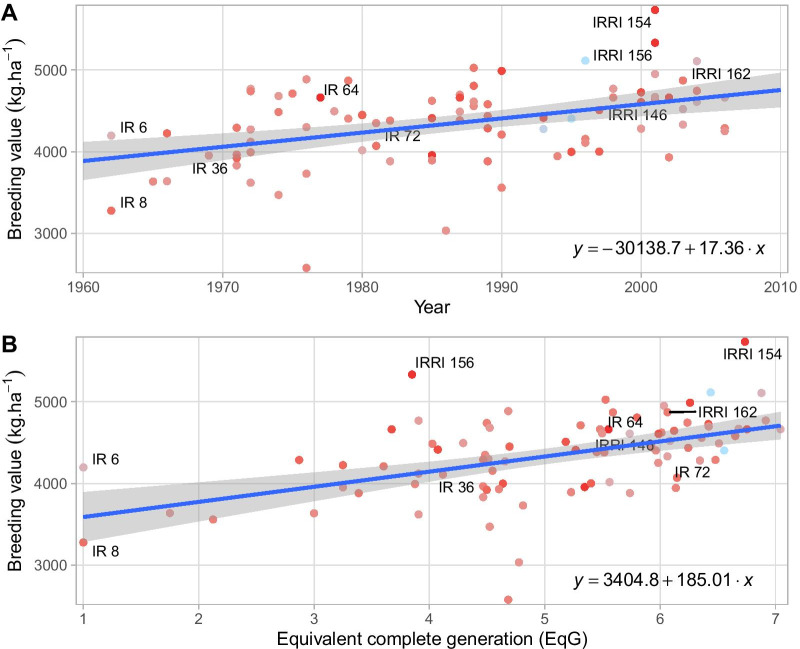
Trend in breeding values for grain yield on 86 IRRI released varieties. **A** Evolution of breeding value according to the year the cross was initially made. **B** Evolution of breeding value according to equivalent complete generation (EqG) as the proxy of realised breeding cycles. The color gradient for the dots represent the reliability of the breeding values from low (blue) to high (red). The blue lines represent the linear regression of the breeding value on the year or EqG. The associated equations are also displayed

Eighty-six released varieties included in the dataset were analyzed separately to better characterize the long-term trend in breeding values for yield and its relationship with EqG (Fig. 3). This includes material dating from the Green Revolution and post-Green revolution eras (IR8, IR36), mega-variety (IR64) and more recent high performing releases (IRRI 154, IRRI 156). Altogether, these lines covered a large period from 1962 to 2006. In this period genetic gain for grain yield was estimated based on released varieties to be 17.36 kg·ha^−1^ year^−1^ (0.46%; Fig. 3A). When regressing breeding values on EqG we observed significant correlation and estimated the rate of genetic gain per cycle to be 185 kg·ha^−1^ cycle^−1^ (4.95%; Fig. 3B).

### Retrospective Analysis of Crosses

**Fig. 4:**
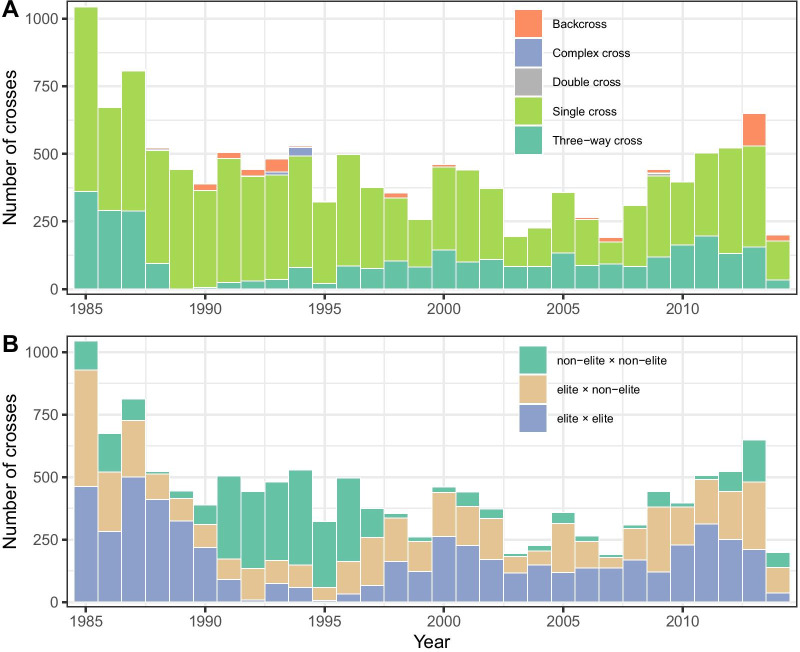
Evolution of the crossing strategy between 1985 and 2014 for IRRI’s irrigated breeding program. The cross type (**A**) and cross status (**B**) are represented. The cross status has been defined based on the equivalent complete generation (EqG) of the two parents (see “Materials and Methods” section)

The pattern of parental selection among crosses made by IRRI’s irrigated breeding program was analyzed over a period of thirty years (1985–2014) to assess the evolution of the crossing strategy and its relationship to EqG. During this period, 13,190 crosses were made. The number of crosses and the proportion of the type of cross varied substantially from one year to the next (Fig. 4A). However, the total number of crosses has been on a downward trend. During this period, most crosses were single crosses (71.9%) or three-way crosses (24.9%) and a small proportion were backcrosses (2.7%), complex crosses (0.4%) or double crosses (0.1%). The proportion of single crosses varied from 42.9% in 2007 to a high of 99.6% in 1989. To further dissect the impact of parental selection and mating design on EqG, crosses from this period were classified as elite by elite (41.4%), elite by non-elite (34.2%) and non-elite by non-elite (24.4%) based on the EqG of the parents. Since an EqG of 4 represented lines from the most advanced available breeding cycle in 1985, any line with an EqG of four or greater was considered elite and any line with an EqG of less than four was considered non-elite. Similar to cross type, the three classes of cross varied substantially from one year to the next (Fig. 4B). Notably, from 1991 to 1997 the proportion of non-elite by non-elite crosses increased dramatically with up to 82% of the crosses falling in this category for that period. This corresponds to a decrease in EqG for the same period and is likely a function of the introduction of new material into the breeding program to achieve the objectives of the NPT initiative (Additional file 2: Fig. S1). During the period 1985–2014, 6,228 unique lines were used as parents and most of them were used only once (65.4%). On the other hand, a few lines were heavily used as parents with 90 lines being used more than 40 times each during the 29-year period. As expected, this list included well-known IRRI varieties such as IR36, IR64, IR72, IRRI 104, IRRI 105, IRRI 118 IRRI 123 and IRRI 154 but also included traditional varieties like Kalimonch, Basmati 370, Shen Nung 89–366 and MD-2 used as donors of alleles with particular value. Interestingly, some of the most used parental lines were crossed during several periods with sometimes more than 20 years between their first and last use (IR64, IR72, IRRI 104, and IRRI 105). This prominent reuse of old material serves to lengthen effective breeding cycles despite advancing the pedigree and is likely one of the primary limitations on the historical rate of genetic gain for grain yield.

### Defining the Elite Core Panel (ECP)

**Fig. 5:**
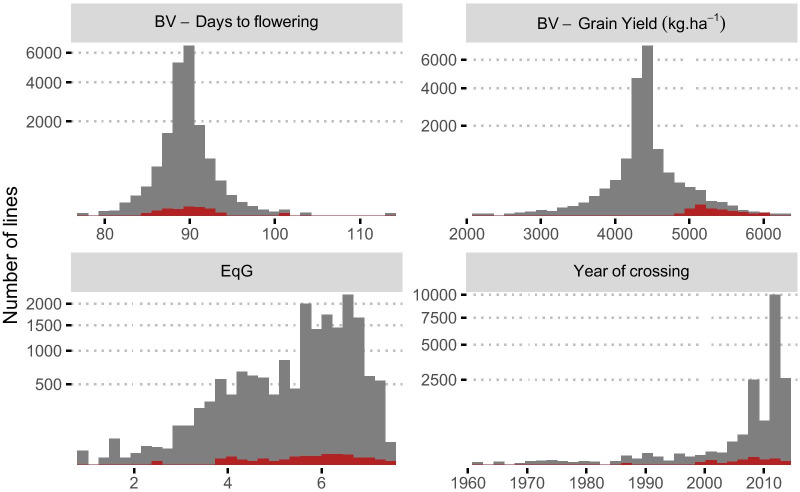
Distribution of 72 elite core panel (ECP) lines among the complete data set. The ECP lines are in red and the rest in grey. Four parameters are represented: breeding values (BV) for days to flowering and grain yield, the equivalent complete generation metric (EqG) and the year the cross was initially made a given line

The best performing lines in terms of breeding value for yield were selected and filtered based on the reliability of the breeding value estimate and their relatedness to other lines in the dataset based on pedigree (see “Materials and Methods” section). The final ECP was composed of 72 lines falling within the top 2% of breeding values, ranging between 4.93 and 6.01 t·ha^−1^ with a mean value of 5.27 t·ha^−1^ (Fig. 5). Most of the selected lines were of medium duration with breeding values for flowering time (days to 50% heading) averaging at 90 days. The average EqG was 5.7 with 90% of the lines having an EqG greater than four. The majority of the lines were developed after 2000 with 37.5% in 2010 and onward (Fig. 5). Interestingly one line (IR05N341) has a significantly lower EqG of 2.44, but has a breeding value of 5158 kg·ha^−1^ and ranks as 45/72 among ECP breeding values. IR05N341 is an NPT inbred with a number of introduced lines in its pedigree, namely SHEN-NUNG 89–366, KETAN LUMBU, GUNDIL KUNING, and JHUM PADDY 7.

### Genetic Characterization of the ECP

**Fig. 6:**
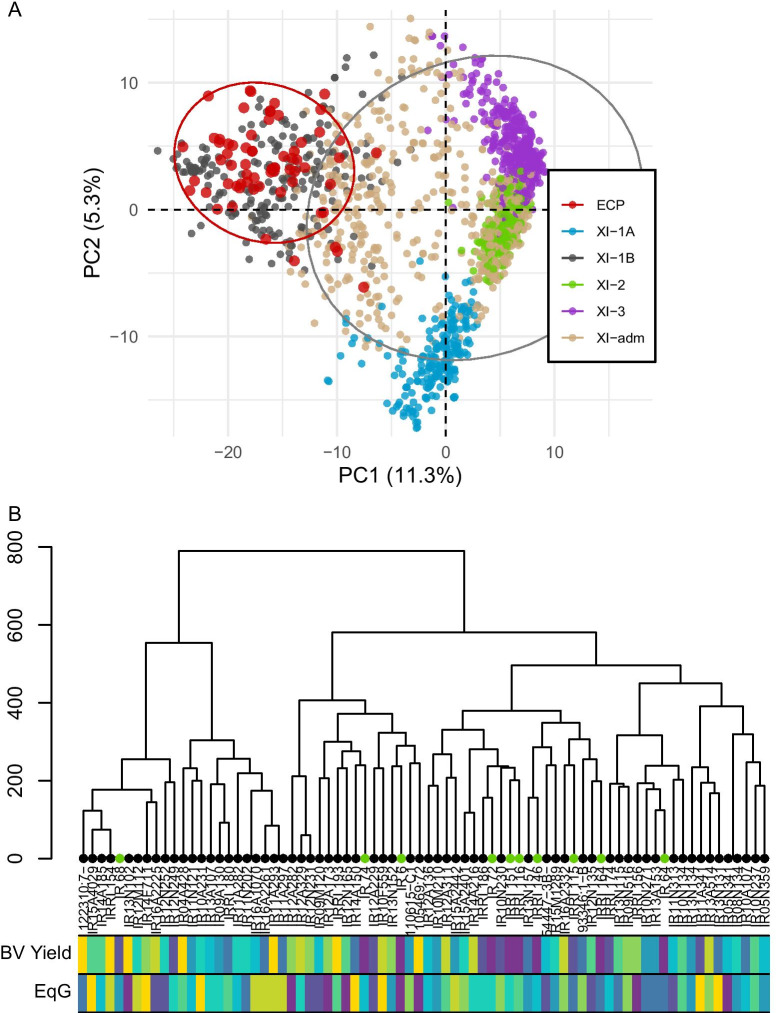
Genotypic characterization of the elite core panel (ECP) based on genome-wide SNP markers. **A** Principal component (PC) analysis showing the grouping of ECP with Xian/indica (XI) group from the 3K-RG lines. **B** Dendrogram showing the relationship between ECP lines and with IRRI varieties (green dots). The heatmap at the bottom represents the breeding value for yield (BV Yield) and the equivalent complete generation (EqG) from the lowest (purple) to the highest (yellow) deciles

In order to quantify the genetic variation available to breeding in the ECP, the panel was genotyped with an amplicon panel of genome-wide markers specifically chosen to be informative among elite indica lines known as the 1k-RiCA (see “Materials and Methods” section). Using publicly available sequence data, the genome-wide SNPs assayed on the ECP lines were compared to the Xian/indica (XI) subpopulation defined by the sequenced 3000 rice genomes (3K-RG) in order to assess the diversity of the elite germplasm relative to a relevant baseline. Principal component analysis revealed that all the ECP lines were mainly clustered in the XI-1B group (Fig. 6A). Not surprisingly, this group includes modern rice varieties from diverse origins with a large representation of material generated by IRRI’s breeding program. Importantly, the selected ECP lines were spread across the entire ‘XI-1B’ group indicating that the selection of ECP lines based on yield performance was still able to capture a large range of diversity within this sub-group. Using linkage disequilibrium measurements from the 1k-RiCA genotype data, the effective population size (*N*_*e*_) was calculated to be 22, indicating a reasonable genetic diversity considering the census population size of 72. Cluster analysis based on genetic distance among ECP lines revealed two main clusters, which further branched into six sub clusters (Fig. 6B). These clusters varied in size (9 to 14 lines per cluster) but all clusters were similar in terms of breeding values for yield (averaging 5.03 to 5.36 tons ha^−1^) and also boasted similar EqG measurements (averaging 5.26 to 6.33) with no significant difference between clusters (*p* value > 0.05).

**Fig. 7:**
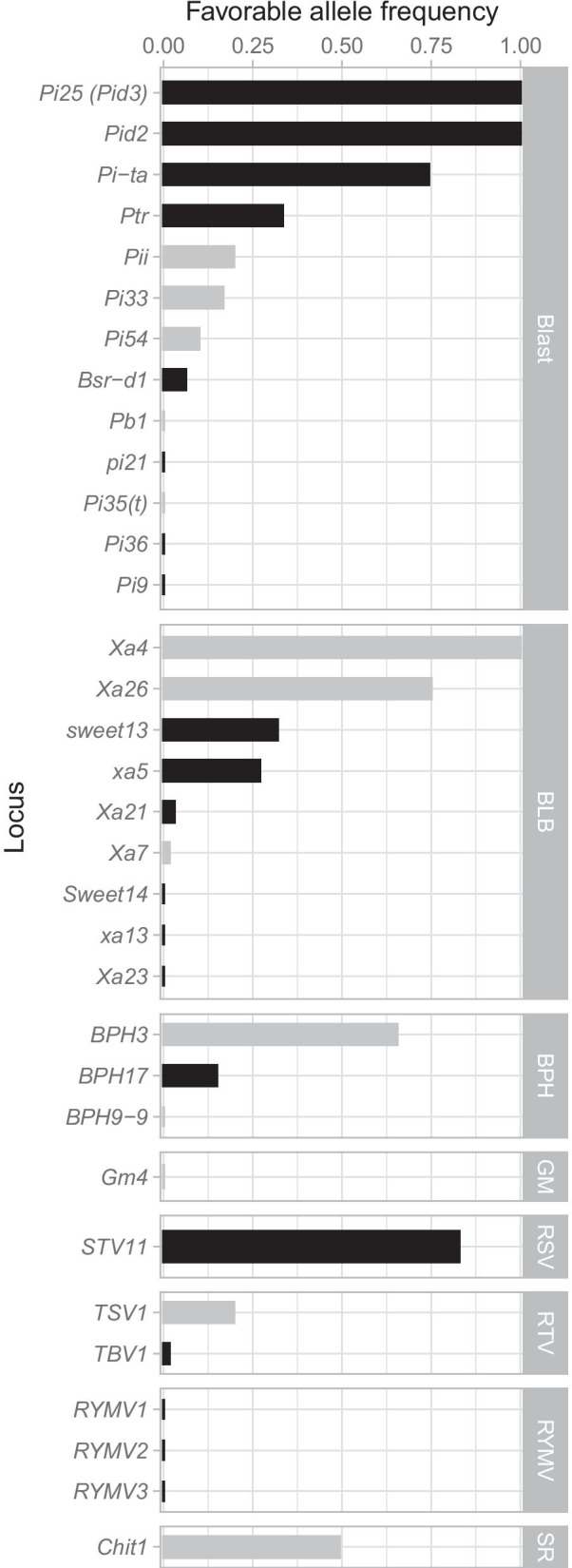
Frequency of favorable alleles in the elite core panel for 33 genes associated with resistance to major biotic stresses. Genes associated with the following pathogens or pests are presented: rice blast, bacterial leaf blight (BLB), brown planthopper (BPH), gall midge (GM), rice stripe virus (RSV), rice tungro virus (RTV), rice yellow mottle virus (RYMV), sheath rot (SR). The color of bars indicates the percentage of genotypes available to compute the frequency: black more than 95% and grey less than 95%

In addition to genome-wide genetic diversity, we also assessed the allele frequency of 33 high value genes contributing to the resistance to major biotic stresses in rice (rice blast, bacterial leaf blight (BLB), brown plant hopper (BPH), gall midge (GM), rice stripe virus (RSV), rice tungro virus (RTV), rice yellow mottle virus (RYMV), and sheath rot (SR)). Genes assayed display a wide range of frequency, from absent to fixed for the favorable allele/haplotype (Fig. 7). For Blast, the frequency of the favorable allele was high or fixed for three genes (*Pita*—74%, *Pi25*—100% and *Pid2*—100%), moderate to low for five genes (*Bsr-d1*—6.3%, *Pi33*—16.7%, *Pi54*—10%, *Pii*—19.7% and *Ptr*—33.3%). For the remaining genes (including *Pi9*, *Pi35, Pi21*) the favorable allele was absent from the ECP. For BLB, *Xa4* (100%) and *Xa26* (75%) presented high allele frequency but with some uncertainty due to missing data. *Xa5* and *sweet13* were also evidenced in the ECP with frequencies of 26.9% and 31.8%, respectively. *Xa21* and *Xa7* were also present but very low frequencies (below 3%). *Xa13, sweet14, Xa23* were absent in the ECP. Concerning BPH, only BPH17 (15%) and BPH3 (65.5%) were found in the ECP. Finally, favorable alleles for *STV11*, *TBV1*, *TSV1* and *Chit1* were also present in the ECP.

### Phenotypic Characterization of the ECP

#### Blast Disease Screening

The ECP lines were evaluated for their level of resistance against five isolates of *Magnaporthe oryzae* under controlled conditions. Based on phenotypic measurement, the most virulent isolate was M64-1-3-9-1 and the least virulent was CA89. A wide variation in the resistance to the five blast isolates was found in the ECP with most of the genotypes displaying intermediate resistance to one or more isolates (Additional file 2: Fig. S2). Not surprisingly, the most resistant genotypes to one isolate were usually not the most resistant for the others suggesting specific isolate-host interactions (Additional file 2: Fig. S3). As the specific combination of favorable alleles at one or several genes associated with blast resistance are likely the primary drivers of phenotypic variation, the ECP lines were classified into groups according to their genetic profile at the surveyed blast genes (Table 2). Genes being either fixed positive (*Pid2, Pid3*) or absent (*Pi9, pi21, Pi35, Pi36*) were excluded from this analysis. No single allele or combination of alleles was found to perform consistently better across the five isolates. However, the presence of *Pi-ta* alone or in combination with *Ptr, Pi5/Pii, Bsr-d1* or *Pi2/Pizt* tended to be associated with more resistant phenotypes. For example, the genotype IR12A311 that carries *Pi-ta*, *Ptr* and *Pi5* was found to be the most resistant across the five isolates. The combination of *Pi-ta* and *Pi2/Pizt* was found in IR93346:1-B-13-7-6-1RGA-2RGA-1-B, a line resistant to 4 isolates. Further, six genotypes (IR09N516, IR12A282, IRRI174, IR13N102, IRRI156, IR12A136) among the ten most resistant carried at least *Pi-ta*. IR11A341, which contains Pi33, was also highly resistant to three isolated and was part of the ten most resistant lines across the five isolates. Further characterization of *Pik-h* and *Pik-m* would be useful to refine the classification of the lines and understand the pattern of resistance.

**Table 2:** Response of the elite core panel to blast disease under controlled environment

Class		Number of lines	Average infection score				
			1K81-25	BN111	CA89	M101-1-2-9-1	M64-1-3-9-1
Susceptible checks		2	4.9	3.8	3.8	4.5	4.8
ECP	Only fixed favorable alleles	18	2.9	2.2	1.4	2.2	2.8
ECP	Ptr	2	2.4	0.5	0.5	1.3	2.3
ECP	Pi33	1	3.5	1.1	0.5	2.1	0.0
ECP	Pi-ta	18	2.5	2.6	1.2	1.6	2.5
ECP	Pii	2	1.6	2.4	1.1	2.4	4.3
ECP	Pi-ta + Ptr	9	2.7	1.5	1.5	1.3	2.9
ECP	Pi-ta + Pii	2	1.9	1.5	1.1	1.8	2.6
ECP	Pi-ta + Pi9	2	1.9	1.4	1.6	1.3	2.4
ECP	Pi-ta + Pi33	2	1.5	2.9	0.7	2.1	2.2
ECP	Pi-ta + Bsr-d1	2	1.5	2.8	0.6	1.2	2.9
ECP	Pi-ta + Ptr + Pii	3	2.0	1.8	0.9	1.1	2.7
ECP	Pi-ta + Ptr + Pi33	1	3.0	1.8	0.6	2.3	1.7
ECP	Pi-ta + Pi54 + Ptr	5	2.8	1.4	0.8	1.7	3.7
ECP	Pi-ta + Pi33 + Pii	1	2.6	1.6	0.7	0.9	3.2
ECP	Pi-ta + Pi33 + Bsr-d1 + Pii	2	2.5	2.2	1.4	1.3	2.4
ECP	Pi-ta + Pi54 + Ptr + Pii	1	3.9	1.5	0.7	1.7	2.1

The disease scores (from 0 (resistant) to 5 susceptible) are categorized by isolate and grouped by allele classes for known resistance genes. Five different isolates of *Magnaporthe oryzae* were used

### Bacterial Leaf Blight Disease Assessment

ECP lines were also screened for resistance to bacterial leaf blight infection against 14 known isolates. The two most virulent isolates were PXO 340 and 99 with an average lesion length of 21.9 cm and 21.3 cm, respectively (Additional file 2: Fig. S4). The least virulent isolate was PXO 61 with most of the lines displaying few symptoms (average lesion length of 2.4 cm). As expected, the ECP lines displayed a large phenotypic variability compared to the checks (IRBB lines or the susceptible check; IR 24). Resistant checks were found to display consistently lower symptoms than the majority of ECP lines (Additional file 2: Fig. S4). Unlike with blast, the response between isolates was significantly correlated with values ranging from 0.26 to 0.77 (Additional file 2: Fig. S5). Similar to blast analysis, the presence of a favorable allele for one or several genes associated with BLB resistance was used to classify ECP lines into groups (Table 3). The *Xa4* allele was present in most of the ECP material. However, it (alone or in combination with *sweet13* and/or *Xa26*) did not significantly reduce the symptoms compared to the susceptible check (IR 24) or the ECP lines without a known favorable allele for BLB genes (Table 3). Favorable alleles for *sweet13* or *Xa26* alone did not reduce the severity of the symptoms compared to the check. The presence of *xa5* alone or in combination with or other genes conferred a better resistance to most of the isolates (except PXO 99 and to a lesser extent PXO 340). *Xa7* (one genotype) and *Xa21* were also found in more resistant genotypes across most of the isolates. The five most resistant ECP genotypes across the 14 isolates, with similar values to the IRBB checks, were IR15A4029 (*Xa4*, *xa5*), IRRI154 (*Xa4*, *xa5*, *Xa26*), IR12N252 (*Xa4*, *xa5*) IR 100,097-B-B RGA-B RGA-8 (*Xa4*, *xa5*, *Xa26*, *sweet13*) and IR12N249 (*Xa4*, *xa5*, *sweet13*).

**Table 3:**
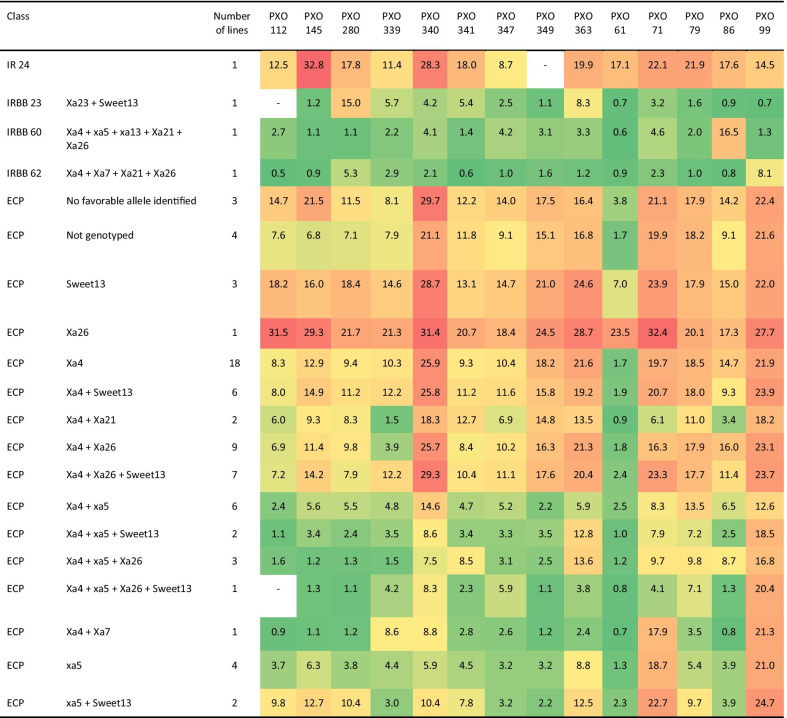
Response of the elite core panel to bacterial leaf blight under controlled environment

The lines were categorized using alleles for known resistance genes. The average lesion length in response to 14 different isolates of *Xanthomonas oryzae pv. oryzae* are displayed. The color gradient depicts the level of resistance of the genotypes: green (short lesion) to red (long lesion)

### Agronomic Performance in Target Environments

**Fig. 8:**
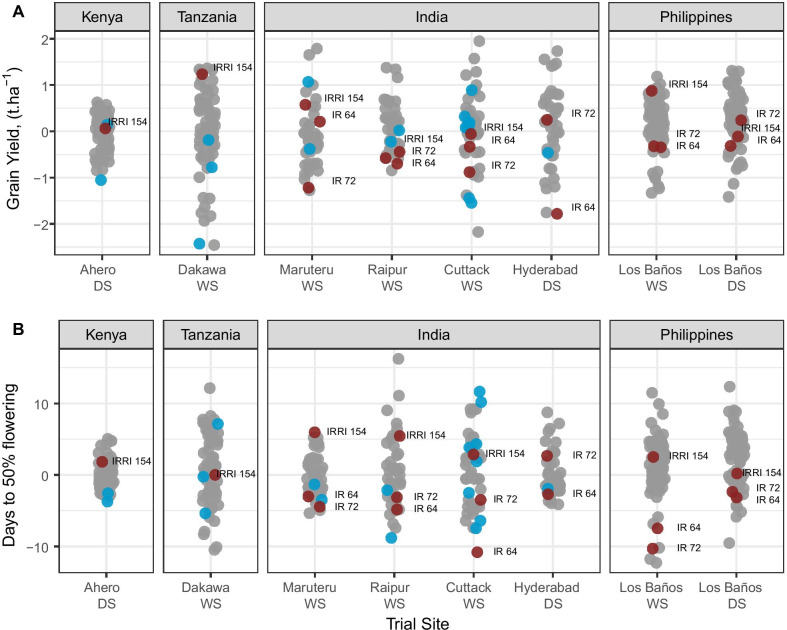
Agronomic performance of the elite core panel in multi-location field evaluations. The panel **A** shows the distribution of the best linear unbiased predictor (BLUP) for grain yield and the panel **B** shows the distribution of BLUPs for days to flowering. The BLUP values are computed for all entries in each field trial using a linear mixed model (see “Materials and Methods” section). The lines composing the core panel are in grey, local and global checks are represented in blue and red, respectively. The name of global checks is displayed next to its BLUP value. The season (WS: wet season or DS: dry season) is also provided along with the location

Phenotypic characterization of the ECP lines was conducted in eight locations to evaluate the agronomic performance of each line in several of the target environments for the IRRI breeding program (Table 4, Fig. 8). To standardize the comparison, agronomic performance of the ECP lines in the field was compared with the three IRRI checks (IRRI 154, IR 64, and IR 72) as well as with a few local checks commonly grown in each region. In the Philippines, IRRI 154 is used as a local check with high yield potential, and an IRRI Check, and is part of the ECP. The repeatability of the trial was good with values ranging from 0.45 to 0.97 for days to flowering and from 0.29 to 0.97 for grain yield. As expected, the performance of ECP lines compared to the local checks was influenced by the environment as genotype by environment interactions are relatively high for traits like grain yield (Additional file 2: Fig. S6). In four environments, ECP lines presented a better grain yield on average compared to local checks (*p* value < 0.05, Table 4). In the remaining environments, ECP lines did not perform significantly better than local checks on average. However, in all environments the variance among ECP lines included some genotypes that presented better grain yield than local checks. These results highlight that despite the Philippines-specific data used to select ECP lines, the material remains relevant to extra-Filipino environments and confirms the importance of this panel for the global breeding program.

**Table 4:** Summary of agronomic trials conducted in target environments to evaluate the performance of the elite core panel

Country	Location	Year	Season	Num of ECP lines tested	Num of local checks	Single Trial H^2^		*p* value relative to local checks
						Days to flowering	Grain yield	
India	Cuttack	2019	Wet	38	8	0.95	0.9	0.001*
India	Hyderabad	2019	Dry	38	1	0.76	0.66	NA
India	Maruteru	2019	Wet	38	2	0.74	0.87	0.282^NS^
India	Raipur	2019	Wet	38	2	0.92	0.55	0.004*
Kenya	Ahero	2019	Dry	67	2	0.45	0.29	0.187^NS^
Philippines	Los Baños	2019	Wet	72	3	0.74	0.67	0.096^NS^
Philippines	Los Baños	2019	Dry	72	3	0.87	0.76	0.008*
Tanzania	Dakawa	2020	Wet	66	3	0.97	0.97	0.041*

* or NS indicate if the p-value is significant (*) or not (NS) at 5%

## Discussion

### Leveraging Historical Data to Estimate Breeding Values

This paper presents a brief but systematic review of the six decades of rice breeding for irrigated environments conducted at IRRI since the green revolution. During this time, the drivers of genetic improvement strategy understandably changed as technology, scientific advancement, and funding priorities evolved. While yield gain was the primary outcome of the Green Revolution breeding strategies, the post-Green Revolution era focused more keenly on changes in plant type, grain quality, biotic and abiotic stresses as well as grain yield using a variety of breeding methods (Fig. 1; Peng et al. 1999, 2008; Khush 2001; Peng and Khush 2003). The historical pedigree information available through the International Crops Information System (ICIS) database (Bruskiewich et al. 2003; McLaren et al. 2005; Portugal et al. 2007) permitted the tracking of crosses and the development of new breeding lines back to 1960 and was a powerful resource for making a data driven and quantitative characterization of breeding methodologies. When utilized alongside newer databases for phenotypic information (Collard et al. 2019) the pedigree data allowed the determination of an individual’s breeding value by integrating the correlated response of relatives harboring the same alleles into the analysis (Piepho et al. 2008). This allowed for a ranking of all the available germplasm using an additive metric of genetic merit and generating a better criterion for parental selection than adjusted phenotypic means alone. While the phenotypic dataset used in the meta-analysis was highly unbalanced (i.e. different lines were evaluated in different locations and years), the mixed model approach is generally robust to such assumptions (Damesa et al. 2017) and creates estimates for fixed effects and predictors for random effects that are unbiased and minimize error variance. The two-stage modeling approach further allowed for the integration of varied experimental designs while the use of the relationship matrix in the second stage of the analysis allowed for the borrowing of information from relatives to further narrow the estimates of uncertainty around an individual's performance.

### The Drivers of the Genetic Gain for Grain Yield

The historical breeding values generated by this analysis provided a convenient mechanism for estimating the historical genetic trend for grain yield in the program. Breeding programs often evaluate genetic gain in many different ways (Rutkoski 2018) and present the result in units that are not always easily interpreted. Most often, this takes the form of a percentage. Here, to aid interpretation, we report genetic gain as kilograms per hectare per year or kilograms per hectare per cycle. The percentages these levels of gain represent relative to the initial performance are given parenthetically for context.

The realized genetic gain for the irrigated program calculated by regressing all 15,286 lines on the year of their origin (year the cross was made from which they were derived) was estimated to be 8.75 kg·ha^−1^ year^−1^ (0.23%) from 1960 to 2014. The estimated rate of genetic gain when the data set was restricted to only IRRI released varieties was estimated to be 17.36 kg·ha^−1^ year^−1^ (0.46%) and 186.24 kg·ha^−1^ cycle^−1^ (4.95%). Previous reports of genetic gain in this program using an era study with a selection of 7 and 12 released varieties have shown gains of 81 and 75 kg·ha^−1^ year^−1^ (~ 1%), respectively (Peng et al. 2000). Estimates of genetic gain for rainfed and drought stress environments in India have been estimated at 34 kg·ha^−1^ year^−1^ (0.68%) and 25 kg·ha^−1^ year^−1^ (0.87%) using 214–242 advanced breeding lines (Kumar et al. 2021). The Brazilian rice breeding program for upland rice has also reported low gains for grain yield with mean gain of 19.1 kg·ha^−1^ year^−1^ (0.67%) over a 26-year period using a meta-analysis of 376 advanced breeding lines (Breseghello et al. 2011). However, in the last decade of their analysis (2002 to 2009), the trend showed an increase in the rate of genetic gain to 45.0 kg·ha^−1^ year^−1^ (1.44%). Similar estimates for irrigated rice in Brazil were reported using rapid-cycle recurrent selection and data from 667 selection candidates that were progeny tested in different breeding cycles (766 kg·ha^−1^ cycle^−1^; 1.98% per year; Morais Júnior et al. 2017). Interpreting the drivers of genetic trend is not simple and speculation in the absence of a complete record of activities can often be misleading. As such, it is helpful to focus on long term patterns in the data. The steeper positive slope of the genetic trend for yield that emerges when only released varieties are considered is consistent with previous reports and can be considered a strong indicator of the positive impact the breeding program has had over time as it has identified and commercialized superior genotypes. The tenfold difference between the per year and the per cycle estimates of genetic gain demonstrates that selection for improved yield has been highly effective on a per cycle basis. This selection response indicates that adequate levels of genetic variance for yield, adequate intensity of selection, and reasonable values for heritability have been maintained during the post-Green Revolution era. The high correlation between breeding values for yield among released varieties and the estimated equivalent generations indicates that cycle time (as measured by EqG) is an important driver of the observed genetic trend. This is consistent with the well-established relationship between generation interval and response to selection (Cobb et al. 2019b). While informative, specific subsets of the breeding germplasm can bias and increase the uncertainty around genetic trend estimates. Using all 15,286 lines for which digitized phenotypic data exists provides a much stronger foundation for assessing base-line rates of genetic gain than potentially interpretable but arbitrary subsets of the data. As this metric incorporates all breeding material (including historical discards), it is not an effective measure of the genetic gain in commercial releases but can be useful for evaluating the impact of breeding innovations on response to selection over cycles.

### Importance of Developing the Elite Core Panel

The contemporary program has moved to a much more intensive recurrent selection strategy based on quantitative genetics principles to drive genetic gain for yield in the context of a disease resistant and high grain quality genetic background. This approach is a natural progression building on previous eras where the focus was on the identification and integration of genetic variation for yield potential traits (Peng et al. 2008). With that, it becomes necessary to systematically evaluate the existing genotypes in the program and select a number of high performing lines to form the basis of a gene pool upon which selection for high breeding value can, in combination with other innovations, drive improved rates of genetic gain (Xu et al. 2017). The 72 lines selected based on breeding value to be part of the ECP essentially represent the initial founders of the recurrent selection program moving forward. While the phenotypic value of this panel should be quickly eclipsed by successive generations of breeding, every new cohort represents an admixture of allelic variance of the panel. Thus, having clearly maintained seed sources for the original lines offers several distinct advantages, including as an elite source of genetic variation to be evaluated alongside the contemporary cohorts for new traits of interest. Such a panel is also helpful for validating trait markers for high-value haplotypes which reduce the occurrence of type I and type II errors when genotyping the progeny (Platten et al. 2019; Cobb et al. 2019a). Once sequenced, the panel also becomes a powerful resource for determining identity-by-descent (IBD) information among progeny cohorts and potentially reducing the need for routine use of high-density markers through the development of a breeding program specific imputation framework (Browning and Browning 2012; Nyine et al. 2019; Wang et al. 2020).

### Genetic Diversity Captured by the Elite Core Panel

A natural concern to limiting the breeding program to crosses among such a small number of lines is the reduction in genetic variation that may occur due both to selection and genetic drift. As the ECP lines were selected based on the pedigree-estimated breeding values, the genetic characterization of the panel is a necessary next step to demonstrate its utility as a resource for breeding in a population improvement program (Warburton et al. 2005; Wen et al. 2012). While the panel itself was selected based on breeding value for yield, the mean flowering time compared to the entire dataset has not changed. This is largely a function of including flowering time as a covariate in the model, which factored out confounding effects due to the positive correlation between yield and flowering in rice.

We used the 3K reference genome panel (Wang et al. 2018) to better understand how well the ECP sampled the genetic space within rice genetic diversity at large. Unsurprisingly, it falls within the Xian/indica 1B group which has been generated through breeding activities in Southeast Asia largely by IRRI (Xie et al. 2015; Wing et al. 2018). Presence of sufficient genetic variation among the ECP lines is further supported by the estimated effective population size (*Ne*) of 22. This value may be underestimated as the markers used for the analysis were specifically designed to be informative in the *indica* subpopulation and harbored high minor allele frequencies on average (Arbelaez et al. 2019). This level of *Ne* is similar to what has been calculated in other rice breeding programs. For example, Grenier et al. (2015) showed a *Ne* in the range of 23–57 in four breeding populations of rice derived through recurrent selection programs. Morais Júnior et al. (2017) observed slightly higher values (40–60) in an irrigated rice breeding population using pedigree data. Values of *Ne* associate positively with additive genetic variation and the ability of a population to respond to selection for the trait under consideration (Falconer and Mackay 1996). Depletion in variability in population is proportional to *Ne*, and the time required to deplete the variability or fix one or other alternative allele in a population is a function of *Ne,* allele frequency (p) in the population, and the selection intensity (Walsh 2003). The theoretical limits of selection response as given by Robertson (1960) postulate that the total response to selection is equal to 2*Ne* times the initial gain in the first generation assuming genes with additive effects and relatively low selection intensity. This is to say that the *Ne* of any given generation is equal to the number of effective cycles before half the genetic variability is eroded by selection or drift. Therefore, holding the unlikely assumption that no new introductions were to be made into the program moving forward, the ECP could theoretically support at least 22 breeding cycles before half of the genetic variance is eroded. This could be further extended by the implementation of marker-optimized mating designs such as optimum contribution selection (Akdemir and Sánchez 2016; Akdemir et al. 2019) and targeted pre-breeding activities.

### Expected Performance of the ECP Across Target Environments

Many traits in rice are governed by a number of high value, large effect alleles that affect patterns of phenotypic variance across environments (Wei et al. 2021). Some of these alleles (particularly disease and grain quality loci) are extremely valuable and deserve proactive management of their frequencies (Cobb et al. 2019a). Understanding these allele frequencies among the ECP lines is therefore essential for setting breeding strategy. As might be expected, the ECP displayed a wide range of frequencies for major pathogens and pests related genes. A few were essentially fixed for the positive allele; many of these represent indica/japonica differences, where the indica allele is favorable, such as *Pi25* or *Pid2*. The value of these genes has already been captured by the breeding program, thus further improvement of these traits must rely on other genetic variation. Other genes are absent, such as *xa13, Xa23,* the rice yellow mottle virus resistance genes, among others. The lack of these genes in elite material necessitates some pre-breeding effort to introduce them to the elite pool before their value can be leveraged (Cobb et al. 2019a). Between these two extremes are the genes that can actually be selected in existing breeding material, and so are those contributing to variation in the elite pool. These include genes such as Xa7, *Xa21 or TBV1* which although present are very rare and only available from particular lines, thus delaying their full deployment as the program generally cannot risk bottleneck a cohort through just a few lines. A few genes are at appreciable frequencies (but not fixed), and so represent diversity that is easily selectable in the existing breeding populations; these include *Pita, Ptr, Pii, BPH32, sweet13, xa5 and TSV1*.

Given the importance of bacterial blight and blast resistance specifically to germplasm exchange across Asia and Africa we decided to challenge the ECP lines against several common isolates of both pathogens to check the effectiveness of the gene combinations present in the ECP against blast and BLB disease in these specific genetic backgrounds. It has been reported by Shanti et al. (2010) that a four-gene combination (*Xa4, xa5, xa13* and *Xa21*) is the most effective combination conferring broad-spectrum resistance for bacterial blight. While 30% of the ECP lines contained one or more bacterial blight resistance alleles, none of the ECP lines contained this specific combination as *xa13* in particular is at a frequency of zero. However, it is clear the gene combinations that are present offer resistance to all tested isolates except PXO 99. PXO 99 is a common *Xanthamonas* isolate in the Philippines (Tu et al. 2000) indicating that while the ECP lines are directly useful in many geographies, a targeted backcrossing and MAS approach is needed to increase the frequency of high value alleles currently at low frequency among the breeding progeny of the ECP. Likewise, the blast resistance genes present in most of ECP lines also effectively controlled the manifestation of disease among the ECP lines for the five strains tested, likely due to the high frequency and broad spectrum resistance offered by the *Pi-ta* locus (Jia et al. 2016).

It is not unexpected that a subset of the breeding germplasm selected based on breeding value for yield would require pre-breeding/backcrossing and MAS to fully address the complexity of trait targets in the product concept. In order to avoid further erosion of the genetic variance (particularly in the form of selective sweeps around low frequency loci) some strategic cautions are warranted. Product development is the primary goal of a breeding program, however when product development is strongly emphasized, it is tempting for breeders to overuse specific high-value lines in a crossing block, at the expense of gene pool management. While every cross may not bring together the complete package necessary for a product release, specific crosses generated with the intention of creating progressively improved allelic combinations (and slowly increase in frequency of major genes) can generate useful lines that can be recycled into the crossing block as parents. Likewise, the use of haplotype-matched backcrossing donors of key genes can be a powerful tool for introducing novel alleles that are at zero or low frequency into the elite gene pool while preserving the availability of genetic variance immediately around them for recombination and the improvement of quantitative traits.

Managing the deployment of single genes within the breeding program due to limitations imposed by their frequency within the ECP is not the only consideration future breeding efforts based on this germplasm resource must consider. Since quantitative traits are not governed by single genes, location main effects and genotype by environment interactions must be routinely accounted for in phenotypic analysis strategies to factor out their strong influence on phenotypic outcomes. Because the primary source of data for determining the breeding values for yield that were used to identify the ECP were trials conducted in the Philippines, an understanding of yield performance in other target environments was also necessary. In order to evaluate the performance of the ECP lines relative to local and IRRI checks in relevant geographies outside the Philippines, six breeding trials were conducted in India, Kenya, and Tanzania. BLUP values centered on the mean performance of each location indicate strong performance of the ECP lines relative to the highest yielding local checks in each location. The performance relative to IRRI varieties and the local checks is a strong indication that the observed genetic variance is manifested as phenotypic variance within each location, indicating that crossing and selection among high performing lines within each breeding zone is likely to result in genetic gain for yield. Further analysis of genotype x environment interactions and the genetic correlations between target environments is warranted to help identify a global testing strategy that best leverages limited public resources available to the IRRI breeding program.

## Conclusion

Achieving short- and medium-term genetic gains for yield is a key target for almost every breeding program. In the case of the IRRI’s breeding program for irrigated systems, the rate of genetic gain for grain yield was estimated at 17.36 kg·ha^−1^ year^−1^ (0.46%) for released varieties. This rate of gain appears to be largely limited by long cycle times and the re-introduction of old material or landraces into the elite pool. This observation highlights the need to optimize the breeding strategy for quantitative traits by using quantitative genetics principles to get closer to the annual 1.5% gain in grain yield needed to cover the expected increase in rice consumption. The elite core panel identified and characterized in this study is a key component of this optimization. Indeed, recurrent selection with short cycles based on elite-by-elite crosses implemented at IRRI to deliver a higher rate of genetic gain for grain yield requires careful management of the genetic diversity, which starts with a comprehensive characterization of the most elite germplasm.

## Materials and Methods

### Historical Yield Data

#### Experimental Studies and Pedigree Information

All the yield data from trials conducted by the irrigated breeding program during the period 2012–2016 across multiple locations were retrieved from the IRRI database. From these trials, the following phenotypic information was extracted: plant height, number of days to flowering, grain yield and number of hills per plot. The phenotypic information extracted were filtered based on the following quality criterion: presence of a randomized experimental design, percentage of missing data for grain yield and flowering time lower than 15%, and harvested area greater than 2 m^2^. We considered an environment as the combination of location, year and season. The environments considered varied in experimental designs according to either a row-column, alpha-lattice, augmented randomized complete block, or ordinary randomized complete block design (RCBD). A total of 102 studies were conducted in 23 environments with a total of 17,216 lines from which 15,286 were sorted out as irrigated rice lines data after filtering (Additional file 1: Table S1). All the studies were conducted in the Philippines, 51 studies having been planted during the wet season and another 51 studies during the dry season. The pedigree information for the selected lines was extracted from the IRRI genealogy management system (McLaren et al. 2005) database using custom scripts. The date of the initial cross was also retrieved for all the breeding lines whose crossing year information was available in the database (16,317 lines). The pedigree information was also used to compute equivalent complete generations (EqG) (Boichard et al. 1997; Gutiérrez et al. 2008; Leroy et al. 2013) for each line. EqG for a given line was calculated as follow:

$$EqG=\sum _{i = 1}^{n}\left(\frac{1}{{2}^{{g}_{i}}}\right)$$

where g_i_ represents the number of generations between the line and its ancestor i (one for the parents, two for the grandparents, etc.).

### Estimating Breeding Values for Lines

A two-stage mixed model analysis (Piepho et al. 2008; Smith and Cullis 2018) using grain yield data as response variable was used to estimate the breeding values of each line. The two-stage mixed model analysis was adopted to account for specific experimental design layouts across the environments (Damesa et al. 2017). In the first stage, each trial or environment (combination of location, year and season) was analyzed separately and best linear unbiased predictors (BLUPs) were extracted per environment using the following baseline mixed-model:

1 $$y_{ij} = \mu + g_{i} + \cdots + \varepsilon_{i}$$

where y_*ij*_ represents grain yield for *i*th observation, μ is the overall mean, g_i_ is the random effect of *i*th genotype with iid g_*i*_ ∼ N(0, Iσ^2^_g_) where σ^2^_g_ is genetic variance and ε_*ij*_ is the residual error with iid ε_*ij*_ ∼ N(0, Rσ^2^_ε_). To account for heterogeneous error variance caused by differences in the numbers of hills harvested from plot to plot and from trial to trial, the diagonal of R was set to *h*/*h*_*max*_ where, *h* is the number of hills harvested and *h*_*max*_ is the maximum number of hills harvested in the environment. The … in the model denotes the blocking factors and a covariate for missing hills which were conditional to the trial. These terms were included in the model because they were identified as improving model fit during analyses of individual trials. Blocking factors were considered random if they had more than five levels. The possible blocking factors were modelled to determine which factors led to the lowest Bayesian information criterion (Spilke et al. 2010; Piepho et al. 2015). For trials that followed a row-column design, the possible factors were row and column, for those following a partially replicated design, the possible factors were row, column, replicate, and block, for those following a RCBD or augmented RCBD, the possible factor was replicate, for those following an alpha-lattice design the possible factors were replicate, block nested within replicate, row, and column. The model with lowest Bayesian information criterion was selected and used to extract BLUP of each line and their prediction error variances (PEV) were obtained for each environment. Reliabilities of the BLUPs were estimated according to $r^{2} = 1 - \frac{PEV}{{\sigma_{g}^{2} }}$. The process for BLUP estimation per environment was repeated for days to flowering.

In the second stage model, the BLUPs obtained from the first stage model were de-regressed by dividing by the reliability as described in Garrick et al. (2009), and used as response variable in the second stage pedigree-based mixed model analysis. The de-regressed BLUPs for yield within each environment were modeled according to Bates et al. (2014). The model used is as follows:

2 $$y_{ij} = \mu + g_{i} + e_{j} + \varepsilon_{ijk}$$

where y_ij_ is the de-regressed BLUP of each line in environment j, μ is the overall mean, g_i_ is a random effect of line i with g_i_ ∼ N(0, Aσ^2^_g_) where σ^2^_g_ is the genetic variance and A is the additive genetic relationship matrix based on pedigree, e_j_ is a fixed effect of the environment j, ε_ij_ is the residual error with ε_ij_ ∼ N(0, Rσ^2^_ε_) where R is a matrix proportional to the residual error covariance matrix and σ^2^_ε_ is the error variance. To account for heterogeneous error variance, the diagonal of **R** was 1/r^2^. In the above model yield was adjusted using days to flowering as covariate in the model. The R packages *lme4* (Bates et al. 2015) and *pedigreemm* (Bates et al. 2014) were used to implement the models.

### Assessment of Rate of Genetic Gain

Genetic gain was assessed using breeding values following the procedure reviewed by Garrick (2010). Briefly, for each year, the breeding values obtained were regressed on the year when the cross was made to get the genetic gain trends.

### Retrospective Analysis of Crosses

The pedigree data of breeding lines developed by the IRRI’s irrigated breeding program was obtained from the genealogy management system. All crosses made were retrieved from the database and filtered based on the availability of the following information: whether or not it belonged to the irrigated program and the year when the cross was made. After those filters, only crosses made in 1985 and onward were available. This is related to the absence of clear boundaries between breeding programs before that date. Indeed, even though lots of material has been produced since IRRI inception in 1960, the information of the breeding program was not recorded in the database before 1985 making it difficult to extract relevant information. For each cross the EqGs of the F1 and of the parental lines were computed based on pedigree information. Each cross was then classified based on EqG of the parental lines. Parental lines with an EqG lower than four (mean EqG value of parents used in 1985) were classified as non-elite material and the remaining part as elite material.

### Formulation of the Elite Core Panel

In order to identify the best breeding genotypes (hereafter called elite lines) the lines were filtered based on high ranking for their grain yield breeding values. Genetic relationship for the genotypes was measured by calculating the coefficient of parentage (CoP) based on the pedigree information using R package *pedigreemm* (Bates et al. 2014). Among the 15,286 evaluated lines, 1192 had reliability for the breeding value greater than or equal to 0.4. Then ten percent of these lines were selected to represent elite material. In total, 119 elite lines were selected among which only 80 elite lines were retained based on seed availability. Further based on seed-viability and intellectual property restrictions, some of the lines were removed and 72 elite lines were forwarded for the formulation of the elite core panel (ECP). The 72 ECP lines are reported in Additional file 1: Table S1.

### Genetic Characterization of the ECP

Genetic diversity of ECP and its relationship with the 3000 rice genomes (Li et al. 2014) was assessed. Effective population size (*Ne*) of ECP was calculated using SNP data. Further, frequency of major trait genes was estimated in the ECP.

### DNA Extraction, Genotyping and SNP Filtering

Leaf samples were obtained from the 72 ECP lines and 10 IRRI varieties (IR 6, IR 64, IR 68, IR 72, IR 74, IRRI 115, IRRI 116, IRRI 146, IRRI 151, IRRI 164) at vegetative growth stage (28–35 days old) plants. DNA was isolated and purified according to the modified Cetyltrimmethyl Ammonium Bromide (CTAB) protocol (Aboul-Maaty and Oraby 2019). Genotyping was done using the 1k-RiCA assay (Arbelaez et al. 2019). The 1k-RiCA SNPs were filtered in TASSEL v5.0 (Bradbury et al. 2007) using the following criteria: individuals with more than 15% of heterozygous loci where removed, markers with more than 15% of missing values and a minor allele frequency below 0.05 were removed. After filtering, 703 markers on 76 lines including 66 ECP lines were retained for downstream genetic analyses (Additional file 1: Table S2).

To enable a better characterization of key genes related to biotic stresses, resequencing data was generated on ECP lines. DNA was extracted from mature leaf tissue using the QIAgen DNeasy Plant maxi kit. Sequencing was performed on total genomic DNA on an Illumina Sequel II system (Macrogen, Korea) or a HiSeq 2000 system (Corteva, Hyderabad). Resequencing data was filtered and trimmed for low-quality base calls using standard pipelines, and mapped to the MSU7 build of the Nipponbare genome. Based on sequencing data quality, four lines were discarded. Base calls at specified informative positions were generated using SAMtools (Li et al. 2009) and analysed to generate a call for the allele present at each of 37 specified high-value genes related to abiotic stresses but only 33 had sufficient data to be called. The information related to the 37 genes is available in Additional file 1: Table S3.

### Diversity of ECP and Its Relationship with 3K-RG, and Favorable Frequency Estimation

The relationship of ECP lines with the indica subpopulation of the 3K-RG was assessed using principal component analysis (PCA). First, the physical position coordinates of the 703 filtered 1k-RiCA SNPs were used to extract the filtered set of markers from the 3K-RG using the rice SNP-Seek database (Mansueto et al. 2017). Out of 703 filtered markers, 625 markers were common between the two data sets and used for downstream analysis. Modern varieties coming from IRRI are known to be within the Xian/Indica (XI) subpopulation. Therefore, the 1787 indica accessions included in the 3K-RG representing part of a diversity of *O. sativa* L. ssp. indica were selected. These accessions, representing landraces and varieties predominately from Asia, were classified according to Wang et al. (2018) as: ‘XI-1A’ with 209 lines mostly from East Asia, ‘XI-1B’ with 205 modern varieties of diverse origin, ‘XI2’ with 285 lines from South Asia, ‘XI3’ with 475 lines from South East Asia, and ‘XI-adm’ with 613 admixed lines. The combined genotypic data of ECP and 1787 indica accessions was imputed in TASSEL with LD-kNNi method using the default parameter (Money et al. 2015) and then formatted as a dosage matrix with marker genotyped coded as 0, 0.5, or 1 (Additional file 1: Table S4). The principal component analysis was performed using the R function *prcomp* (R Core Team 2018). The principal components were extracted and then visualized using the R package *ggplot2* (Wickham 2016).

To assess the diversity and genetic relationships among ECP lines, a hierarchical clustering analysis was performed based on 703 filtered 1k-RiCA SNPs using Manhattan distance and Wards methods with the functions *dist* and *hclust* in R software (R Core Team 2018). Dendrogram was created using the R package *dendextend*.

### Estimation of Effective Population Size (*Ne*)

Effective population size (N_e_) was estimated based on linkage disequilibrium (LD) information between the markers (Hill 1981) using the following equation: $Ne = \frac{1}{4c}\left( {\frac{1}{{E\left( {r^{2} } \right)}} - 1} \right)^{{}}$ (Sved 1971). Here, c is the genetic distance in Morgans and was calculated by dividing the physical distance of each marker by 250 kb, and E(*r*^*2*^) is expected *r*^*2*^ for a marker distance c. Pearson's squared correlation coefficient (*r*^*2*^) of each pair of loci as a measure of LD in relation to physical distance was calculated in the R package *sommer* (Covarrubias-Pazaran 2016).

### Phenotypic Characterization of the ECP

ECP accessions were evaluated for disease resistance to two important pathogens (*Magnaporthe oryzae,* the fungus causing blast disease and *Xanthomonas oryzae pv. oryzae* (*Xoo*), the bacteria causing bacterial leaf blight disease (BLB)) were assessed in controlled conditions. The pathogens for both blast and BLB were obtained from isolates collected in the Philippines. In addition to the evaluations in controlled condition, agronomic traits were evaluated in multi-environment experiments located in the target regions of IRRI’s irrigated breeding program.

### Evaluation of ECP Accessions Against Blast Disease

#### Plant Material

72 elite lines from the ECP, 2 highly susceptible rice cultivars (Lijiangxintuanheigu and CO 39) and four blast resistant checks (IRBLta2-Pi, IRBLSH-B, IRBLkm-Ts and IRBLKh-K3) were evaluated. The experiment was set by planting test genotypes in the screen house trays at IRRI, Los Baños**,** a systematic arrangement was adopted, ten plants were established per genotype and each plant was treated as a replicate for the genotype. The two check lines were planted alternately at intervals of every ten test genotypes. Plant establishment and management were according to the rapid generation advancement protocol (Collard et al. 2017).

#### Blast Strains

Five highly virulent *Magnaporthe oryzae* isolates, M101-1-2-9-1, M64-1-3-9-1, Ca89, BN111 and IK81-25 were selected based on their reported differential disease spectrum on blast monogenic lines carrying blast resistance genes *Pi54*, *Pi9*, *Pi-ta*, *Pi-km* and *Pi2*. These Isolates are part of the set of 20 standard differential blast isolates in the Philippines. They were selected due to their good sporulation and ability to differentiate the differential varieties. Further, they have been used over time in rice variety selection experiments and their pathogenicity has remained stable (Telebanco-Yanoria et al. 2008).

#### Inoculation and Assessment of Infection

Single spore conidial stocks were revived on prunes Gulaman medium. The inoculated plates were incubated at 25 ± 1 °C for 10 days, after which inoculated plates were scraped with a sterilized glass slide and exposed to continuous light for 4 days to induce heavy sporulation. Conidia were dislodged by rubbing the incubated plates gently with a glass slide. The spores were washed with 10 ml sterilized distilled water homogenized with 0.02% Tween 20. The suspensions were filtered through three layers of gauze mesh and concentration adjusted to 10^5^ conidia per ml using a hemocytometer. Plants were inoculated 21 days after planting following the standard methods (Bonman et al. 1986). Seven days later plants were assessed for disease symptoms based on the Standard Evaluation System for Rice (IRRI 2013). Ten plants were observed and scored at a scale of 0 to 5 [score 0 represents absence of blast lesions and graded as highly resistant (HS), 1 = resistant, 2 = moderately resistant, 3 = moderately susceptible, 4 = susceptible, whereas 5 was considered highly susceptible. One line was removed from the analysis due to the high number of missing data. The average infection scores for all the ECP lines and the checks are available in Additional file 1: Table S5.

### Evaluation of Bacterial Leaf Blight Strains Effect on ECP Lines

#### Plant Material

The 72 ECP lines were tested alongside with three checks carrying different BLB resistance genes (IRBB23 (*Xa23*, *Sweet13*), IRBB60 (*Xa4, xa5, xa13, Xa21* and *Xa26*) and IRBB62 (*Xa4*, *Xa7*, *Xa21* and *Xa26*)) and a susceptible cultivar (IR24). The experiment was conducted under greenhouse conditions in systematic arrangement with two replications at IRRI, Los Baños, Philippine in 2018. 28 minoru trays (two trays per strain) were prepared. Crop management was performed according to rapid generation advancement protocol (Collard et al. 2017).

#### BLB Strains

Fourteen Philippine strains (PXO) representing 10 races of *Xanthomonas oryzae pv. oryzae* (*Xoo)* were used, namely: PXO16, PXO86, PXO79, PXO340, PXO71, PXO112, PXO99, PXO145, PXO280, PXO339, PXO349, PXO347, PXO363 and PXO34. The *Xoo* strains were sourced from IRRI laboratory stock and revived using modified Wakimoto’s medium for 48 to 72 h at 28 °C. Inoculum preparation was implemented according to Goto (2012).

#### Inoculation and Assessment of Infection

The accessions were prepared for inoculation by punning the lower leaves and extra tillers on the 35^th^ day after seeding. Plants were inoculated with one strain of *Xoo* on the 45^th^ day after seeding using a leaf-tip clipping method. Three leaves per plant were inoculated. Evaluation of resistance was done at 14 days after inoculation (DAI) by measuring the lesion starting from the point of inoculation to the end with visible symptoms. For a given isolate, the genotypes with less than four measurements (out of six) were removed from the analysis. The average lesion length was computed for all the ECP lines and the checks. The final dataset had 2.9% missing data and is available in Additional file 1: Table S6.

### Field Based Evaluation and Data Analysis

Phenotypic characterization of ECP for grain yield was conducted in Kenya, Tanzania, India and the Philippines. Different experimental designs such as randomized complete block, alpha-lattice and partially replicated designs were used across these environments (Additional file 1: Table S7). Plot-level yields were normalized based on plot size and all the experimental data is stored in the breeding for results (B4R) data management system.

Grain yield (ton/ha) and days to 50% flowering time (days) from all the ECP trials were used in this study. The best linear unbiased predictor (BLUP) values were calculated using the *predict* function from the R package *asreml* in which the entry was used as a random effect in all the trials. For the trials that followed a partially replicated design, the possible factors were row, column, replicate, and block, for those following a randomized complete block design, the possible factor was replicate, for those following an alpha-lattice design the possible factors were replicate, block nested within replicate, row, and column. The BLUP values were used to rank the performance of the accessions along with the IRRI and local checks grown in each location. The three IRRI checks used in this study were IR 64, IR 72 and IRRI 154, which are high yielding varieties released in the years 1985, 1988 (Peng and Khush 2003) and 2010, respectively. The local checks are specific to the regions and are selected by the partners conducting the trial. BASMATI 370 and IRRI 215 in Kenya; SUPA, SUPA BC and TXD 306 in Tanzania; MTU 1010, IGKV-R1, CG-Deobhog, CR Dhan-304, CR Dhan-307, ARIZE 6444 Gold, SWARNA, IRRI 216 in India were the local check entries in the trials. For the Philippines, the global checks also served as the local checks.

## Supplementary Information

**Additional file 1: Table S1.** List of all lines 15,286 of the irrigated breeding program selected for the analysis of genetic gain. The following information are available: designation (name of the lines), GID (identification number), parentage, year of the initial cross, type of entry (breeding lines or IRRI released varieties), ECP (belong or not to the elite core panel), equivalent complete generation (EqG), breeding value for yield corrected from flowering time. **Table S2.** Genotypic data for 66 ECP and 10 IRRI varieties from the breeding program on 703 SNP from the 1k-RiCA platform. The data are in hapmap format. **Table S3.** Information on the 37 high-value genes related to abiotic stresses. The favorable allele frequency is reported for 33 genes which had sufficient data. **Table S4.** Combined genotypic data of ECP and 3K-RG indica accessions formatted as a dosage matrix with SNP genotypes coded as 0, 0.5, 1. **Table S5.** Results of the evaluation of the elite core panel against blast disease. The average infection score to five highly virulent *Magnaporthe oryzae* isolates measured in controlled conditions are reported along with the favorable alleles (if any) for each line. The resistant and susceptible checks are also included. **Table S6.** Results of the evaluation of the elite core panel against bacterial leaf blight. The average average lesion length (based on six plants) to fourteen isolates of *Xanthomonas oryzae pv. oryzae* measured in controlled conditions are reported along with the favorable alleles (if any) for each line. The resistant and susceptible checks are also included. **Table S7.** Studies conducted in the target regions for IRRI's irrigated rice breeding program to evaluate the performance of the elite core panel (ECP) lines in 2019.

**Additional file 2: Fig. S1.** Evolution of equivalent complete generation (EqG) of parental lines used between 1985 and 2014 for IRRI’s breeding program for irrigated systems. **Fig. S2.** Distribution of the infection score (0 = highly resistant to 5 = hihgly susceptible) for five isolates of blast (*Magnaporthe oryzae*). The elite core panel (ECP) lines are in grey, the susceptible checks (Lijiangxintuanheigu and CO 39) are in orange and the resistant checks (IRBLta2-Pi, IRBLSH-B, IRBLkm-Ts and IRBLKh-K3) in green. **Fig. S3.** Scatter plots and rank correlations between blast isolates using infection scores. **Fig. S4.** Distribution of the lesion length of elite core panel (ECP) lines after controlled inoculation with *Xanthomonas oryzae pv. Oryzae*. Fourteen different isolates were used to assess the level of resistance of the ECP lines. The ECP is in grey, the susceptible check (IR 24) is in orange and the resistant checks (IRBB23, IRBB60 and IRBB62) in green. **Fig. S5.** Scatter plots and rank correlations between bacterial leaf blight isolates using the average lesion length for each genotype. **Fig. S6.** Correlation matrix for grain yield (**A**) and time to flowering (**B**) for all the environments where the ICP lines have been evaluated.

## Data Availability

The datasets supporting the conclusions of this article are included within the article and its additional files.

## Abbreviations

BLUP: Best linear unbiased predictions
BV: Breeding values
ECP: Elite core panel
EqG: Equivalent complete generation
MAS: Marker assisted selection
Ne: Effective population
NPT: New plant type
RYMV: Rice yellow mottle virus
SSD: Single seed descent
