# Supplementary material for: Identification of an Elite Core Panel as a Key Breeding Resource to Accelerate the Rate of Genetic Improvement for Irrigated Rice
Source: Rice (N Y). 2021 Nov 13;14:92. doi: 10.1186/s12284-021-00533-5 (PMC8590642; doi:10.1186/s12284-021-00533-5)
Supplement: Supplementary file 2 — Additional file 2: Fig. S1. Evolution of equivalent complete generation (EqG) of parental lines used between 1985 and 2014 for IRRI’s breeding program for irrigated systems. Fig. S2. Distribution of the infection score (0 = highly resistant to 5 = hihgly susceptible) for five isolates of blast (Magnaporthe oryzae). The elite core panel (ECP) lines are in grey, the susceptible checks (Lijiangxintuanheigu and CO 39) are in orange and the resistant checks (IRBLta2-Pi, IRBLSH-B, IRBLkm-Ts and IRBLKh-K3) in green. Fig. S3. Scatter plots and rank correlations between blast isolates using infection scores. Fig. S4. Distribution of the lesion length of elite core panel (ECP) lines after controlled inoculation with Xanthomonas oryzae pv. Oryzae. Fourteen different isolates were used to assess the level of resistance of the ECP lines. The ECP is in grey, the susceptible check (IR 24) is in orange and the resistant checks (IRBB23, IRBB60 and IRBB62) in green. Fig. S5. Scatter plots and rank correlations between bacterial leaf blight isolates using the average lesion length for each genotype. Fig. S6. Correlation matrix for grain yield (A) and time to flowering (B) for all the environments where the ICP lines have been evaluated. [file 12284_2021_533_MOESM2_ESM.pdf]

Supplementary Information

Identification of an elite core panel as a key breeding resource to accelerate the rate of genetic improvement for irrigated rice.

Roselyne U. Juma[1, 2], Jérôme Bartholomé[1, 3*], Parthiban Thathapalli Prakash[1], Waseem Hussain[1], John D. Platten[1], Lopena V[1], Verdeprado H[1], Rosemary Murori[1], Alexis Ndayiragije[1], Sanjay Katiyar[1], M. Rafiqul Islam[1], Partha S. Biswas[1,5], Jessica E. Rutkoski[1,6], Juan D. Arbelaez[1,6], Felister N. Mbute[4], Douglas W. Miano[4], Joshua N. Cobb[1,7]*

[1] Rice Breeding Platform, International Rice Research Institute, DAPO Box7777, Metro

Manila, Philippines

[2] Present affiliation: Kenya Agricultural and Livestock Research Organization, KALRO Non-Ruminant Research Institute (NRI), 50100-169, Kakamega, Kenya

[3] AGAP, Univ Montpellier, CIRAD, INRA, Montpellier SupAgro, Montpellier, France

[4] University of Nairobi, (UON) Upper Kabete Campus 29053, – 00625. Kangemi

[5] Present affiliation: Bangladesh Rice Research Institute, Gazipur, Bangladesh

[6] Present affiliation: The University of Illinois at Urbana-Champaign

[7] Present affiliation: RiceTec. Inc, PO Box 1305, Alvin, TX 77512

* Corresponding authors: Joshua N. Cobb: jcobb@ricetec.com / Jérôme Bartholomé: jerome.bartholome@cirad.fr

1. Supplementary methods

1.1. Addition of landmark lines

Additional landmark lines that contributed most to the pedigree of elite core panel lines were identified and genotyped with the same set of markers. The landmark lines were identified through pedigree tracing using R software. All pedigree generations of the elite lines were traced to identify historical founders. All landraces and segregating lines were excluded, retaining only later generations (F5+). Redundant lines were identified using the coefficient of parentage matrix between the pedigrees. Genetically similar lines were discarded, retaining only one from each group thus selecting 373 lines. A relationship matrix was created between the selected historical lines and the elite lines to ensure distinctness between them. Seed availability for selected historical lines was verified and 10 landmark lines were finally selected.

1.2. Purity testing of the ECP lines

The purity test of the genotypes was conducted for quality control and identification of true to type plants representative of the elite lines. The identified genotypes for the core panel were planted from 21 day old seedlings raised in a wet nursery. There were 72 genotypes, each

genotype was replicated between 10-12 times. Leaves samples were obtained from each genotype in ten replications at vegetative growth stage (28-35 days old) plants. The leaves were lyophilized, DNA was isolated and purified according to the modified Cetyltrimmethyl Ammonium Bromide (CTAB) method (Idress, 2011; Yan et al., 2011). DNA profiling was done on a ten purity SNP chip, SNP calls were by Illumina (Liu et al., 2012). To identify true to type plants, a 90% total snp call threshold was set where reference or alternative snp were identified for the genotype profile. The marker that satisfied the condition was selected for the snp in the profile. Using R-statistical software, a reference profile was identified for each genotype. The snp for each replication were matched with the reference profile to identify the replicates with at least one missing snp. Further, total missing snps per replicate were computed to find total number of missing snp calls and percent missing calls per genotype. The purity of each genotype was checked by identifying the replicate having more than one difference with the reference SNP sequence. The selected plants were maintained to maturity, the seed was amplified and used for genotypic and phenotypic characterization of the ECP lines.

Supplementary figures

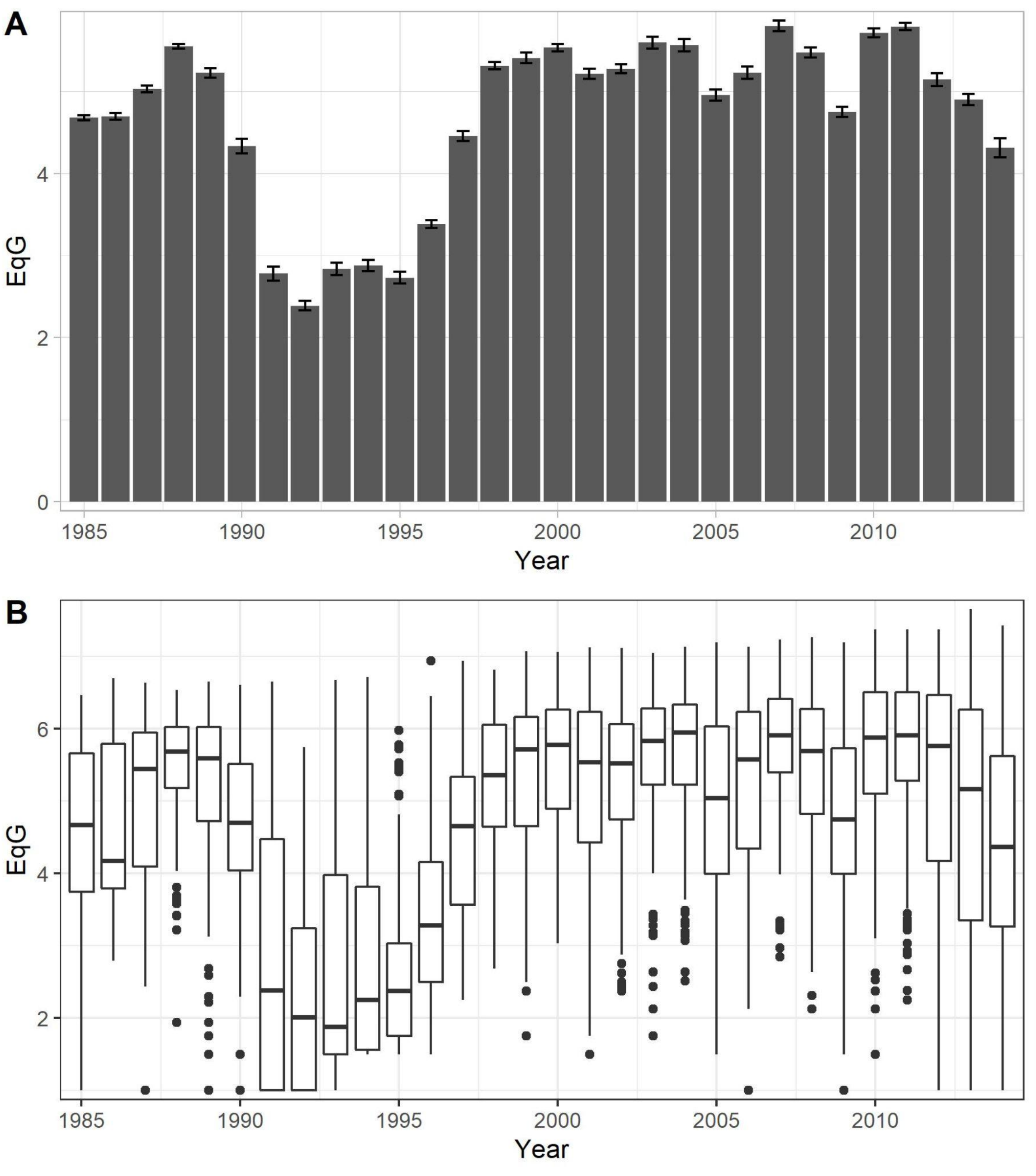

Figure S1: Evolution of equivalent complete generation (EqG) of parental lines used between 1985 and 2014 for IRRI's breeding program for irrigated systems.

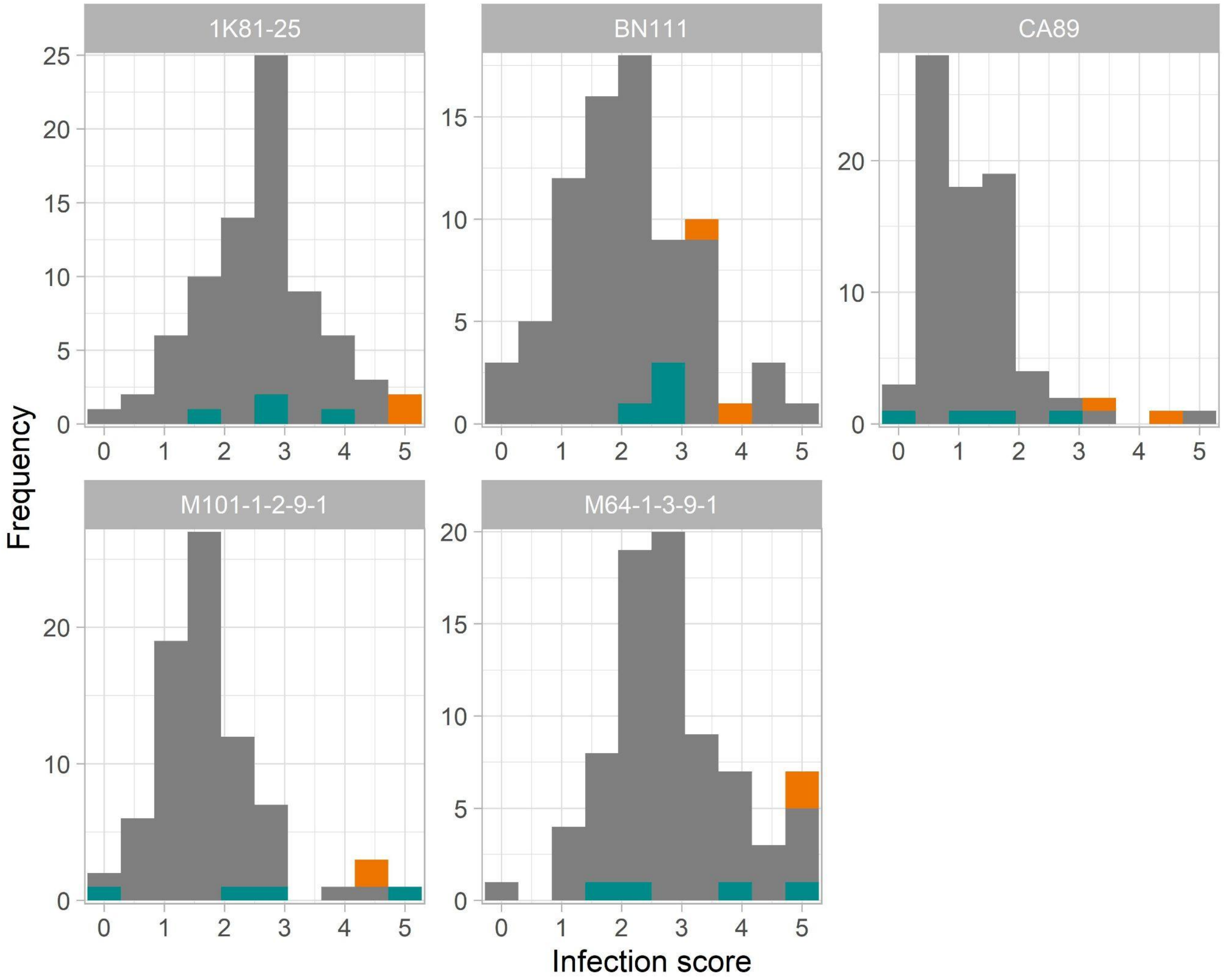

Figure S2: Distribution of the infection score (0 = highly resistant to 5 = highly susceptible) for five isolates of blast (*Magnaporthe oryzae*). The elite core panel (ECP) lines are in grey, the susceptible checks (Lijiangxintuanheigu and CO 39) are in orange and the resistant checks (IRBLta2-Pi, IRBLSH-B, IRBLkm-Ts and IRBLKh-K3) in green.

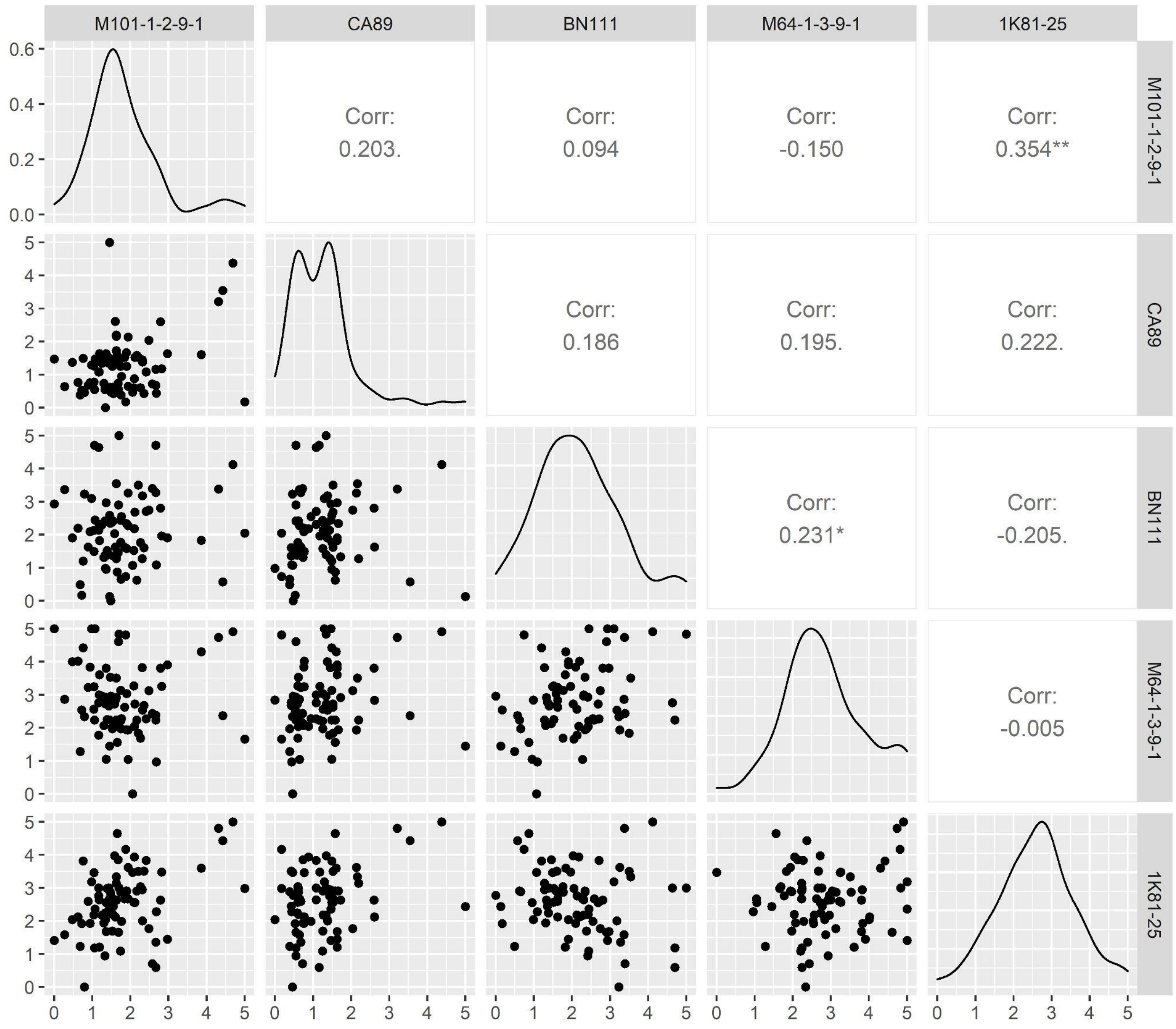

Figure S3: Scatter plots and rank correlations between blast isolates using infection scores.

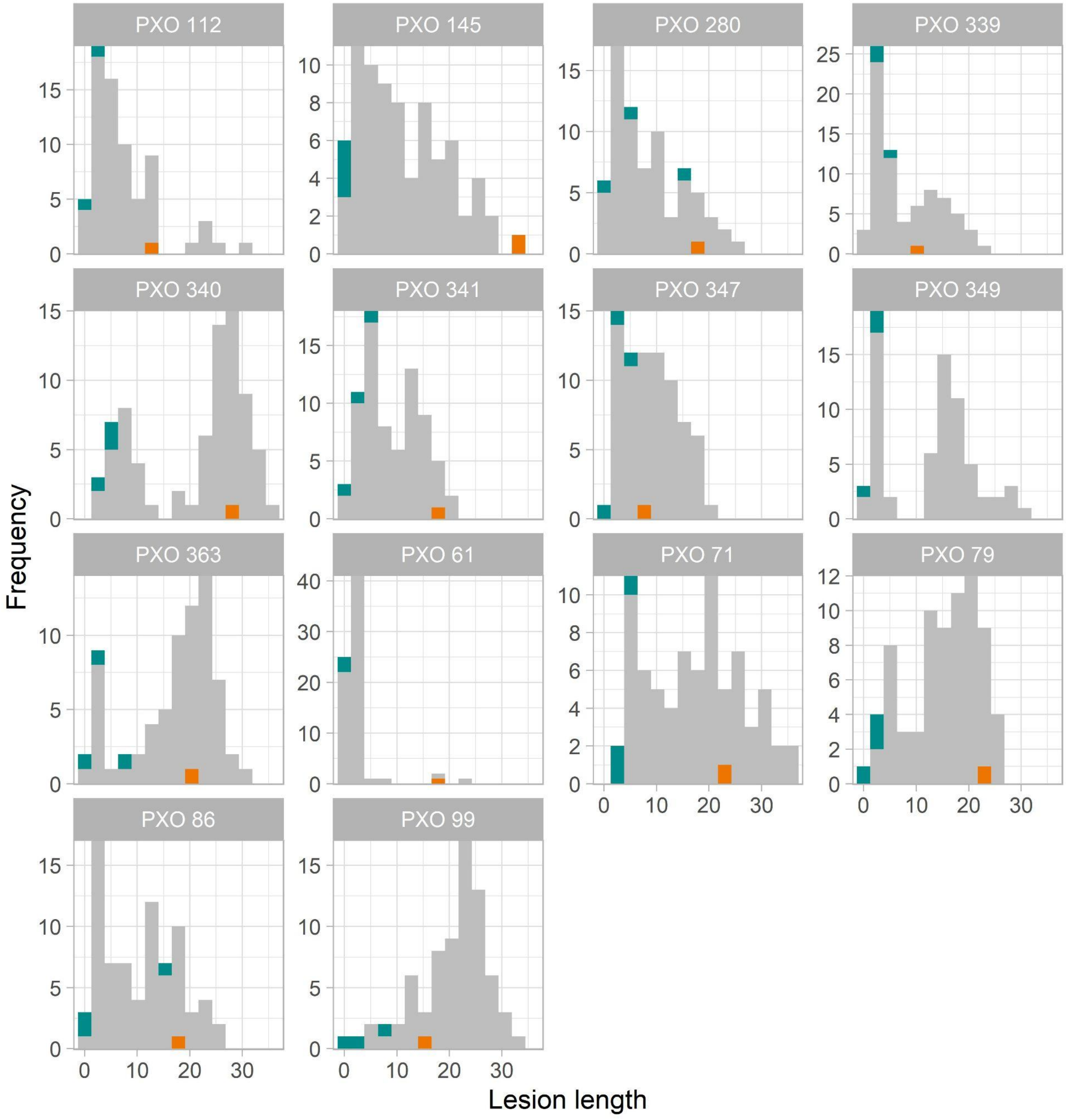

Figure S4: Distribution of the lesion length of elite core panel (ECP) lines after controlled inoculation with *Xanthomonas oryzae pv. Oryzae*. Fourteen different isolates were used to assess the level of resistance of the ECP lines. The ECP is in grey, the susceptible check (IR 24) is in orange and the resistant checks (IRBB23, IRBB60 and IRBB62) in green.

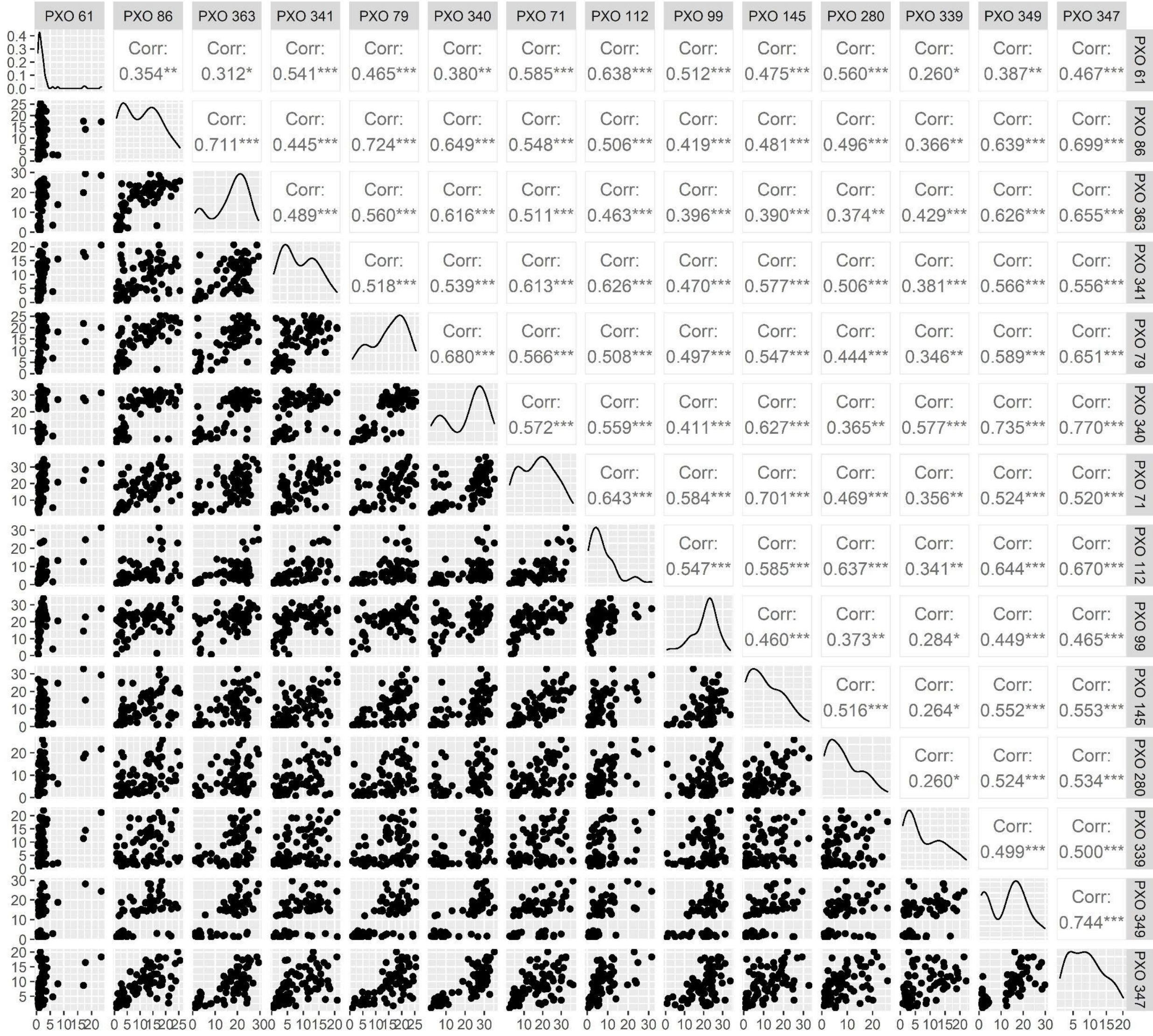

Figure S5: Scatter plots and rank correlations between bacterial leaf blight isolates using the average lesion length for each genotype.

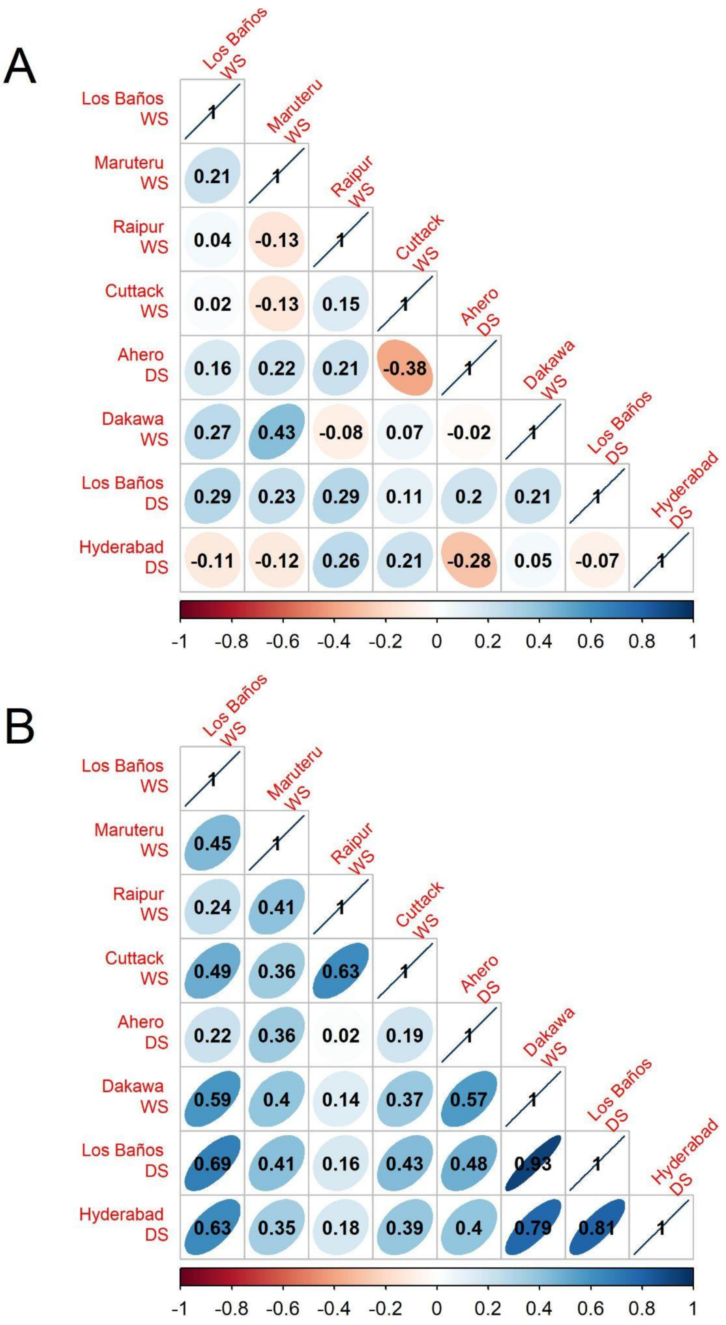

Figure S6: Correlation matrix for grain yield (A) and time to flowering (B) for all the environments where the ICP lines have been evaluated.
